# Using Habitat, Morphological, and Genetic Characteristics to Delineate the Subspecies of Sharp‐Tailed Grouse in South‐Central Wyoming

**DOI:** 10.1002/ece3.71429

**Published:** 2025-05-12

**Authors:** Jonathan D. Lautenbach, Andrew J. Gregory, Stephanie Galla, Aaron C. Pratt, Michael A. Schroeder, Jeffrey L. Beck

**Affiliations:** ^1^ Department of Ecosystem Science and Management University of Wyoming Laramie Wyoming USA; ^2^ Department of Biological Sciences University of North Texas Denton Texas USA; ^3^ Department of Biological Sciences Boise State University Boise Idaho USA; ^4^ George Miksch Sutton Avian Research Center Bartlesville Oklahoma USA; ^5^ Washington Department of Fish and Wildlife Bridgeport Washington USA

**Keywords:** genetics, genomics, habitat, morphology, Sharp‐tailed Grouse, subspecies, *Tympanuchus*

## Abstract

Identifying species and subspecies is the foundation for focusing conservation efforts and studying evolutionary ecology. Subspecies delineation has occurred using multiple data types, including ecological, morphological, and genetic data. There are currently seven recognized Sharp‐tailed Grouse ( *Tympanuchus phasianellus* , Linnaeus, 1758) subspecies, with two of these subspecies occurring in Wyoming: Columbian Sharp‐tailed Grouse (*T. p. columbianus*) and plains Sharp‐tailed Grouse (*T. p. jamesi*). There is a third population of Sharp‐tailed Grouse in south‐central Wyoming with an unknown subspecific identification. Historically, this population has been classified as Columbian Sharp‐tailed Grouse; however, previous genetic evidence questioned this classification. To better understand the subspecific status of this south‐central Wyoming population, our study used habitat characteristics, morphological characteristics, and genetic data (microsatellite loci and single nucleotide variants) collected from known Columbian Sharp‐tailed Grouse, known plains Sharp‐tailed Grouse, and the south‐central Wyoming population of Sharp‐tailed Grouse. We modeled differences among the populations using discriminant analysis of principal components and Random Forests classification models. Across all four datasets and both modeling techniques, we found that each population (Columbian Sharp‐tailed Grouse, plains Sharp‐tailed Grouse, and south‐central Wyoming population of Sharp‐tailed Grouse) generally represented its own cluster. Our results suggest that the population of Sharp‐tailed Grouse in south‐central Wyoming is different from both Columbian and plains Sharp‐tailed Grouse. We recommend further evaluation of the subspecies of Sharp‐tailed Grouse using more targeted phylogenomic studies to identify if Sharp‐tailed Grouse in south‐central Wyoming represent a separate subspecies or are a distinct population of another subspecies. Our results potentially change our understanding of Columbian Sharp‐tailed Grouse distribution and management and highlight the importance of using a more comprehensive approach to identifying subspecies.

## Graphical abstract

Sharp‐tailed Grouse are a species of North American prairie‐grouse comprised six extant subspecies. A population of sharp‐tailed grouse in southcentral Wyoming was historically classified as Columbian Sharp‐tailed Grouse, however, our research suggests that this population does not belong to any of the extant Sharp‐tailed Grouse subspecies. Our research changes our understanding of Sharp‐tailed Grouse subspecies and opens the door to additional research on this topic.

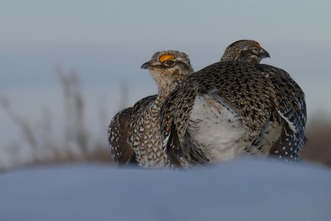

## 1 Introduction

Taxonomic classifications assist ecologists in measuring the distribution of biodiversity on a changing planet and enacting conservation actions. Infraspecific taxonomic classifications, or those below the rank of species (e.g., subspecies), are important in providing designations to conserve biodiversity into the future (Haig et al. 2006; Haig and D'Elia 2010; Winker 2010; Taylor, Perrin, et al. 2017). Subspecies and other infraspecific classifications (e.g., ecotypes, varieties, distinct population segments, and evolutionary significant units) represent the diversity of functional traits—and therefore evolutionary potential—within a species (Haig et al. 2006) and the conservation of subspecific taxa may help ensure species persistence in a changing environment (Winker 2010). This has been explicitly recognized by the Endangered Species Act as amended in 1978 (16 U.S.C. §§ 1532[16]) allowing the listing of not just entire species but also subspecies, Distinct Population Segments, and Evolutionary Significant Units (National Oceanic and Atmospheric Administration [NOAA] 1991; U.S. Fish and Wildlife Service 1996). Despite this recognition of the importance of infraspecific classification of organisms, there is still debate about what methods best describe significant intraspecific variation (Zink 2004; Haig et al. 2006; Remsen 2010; Winker 2010; Patten 2015).

Different approaches have been used to conceptualize and describe subspecies (Haig et al. 2006; Winker 2010). Some of the primary approaches used to describe subspecies have been based on ecology (e.g., the ecological and biological species concepts), morphology (e.g., the morphological species concept), and phylogeny (e.g., the phylogenetic species concept). Each of these species concepts has limitations associated with the application to define subspecies. For example, within the ecological species concept, populations may inhabit different habitats, but may not be distinguishable in terms of genetics or morphology; within the morphological species concept, morphological differences in subspecies may not represent genetic differences (Zink 1989, 2004; Ball and Avise 1992; Haig et al. 2006); and the phylogenetic species concept recognizes species as the smallest supported monophyletic unit, but groupings within species (e.g., subspecies) do not exist (Haig et al. 2006). Because of these issues, some authors suggest using multiple species concepts to define and assess the validity of subspecies (Helbig et al. 2002; Haig et al. 2006; Wallin et al. 2017), though some recent studies have used or suggested the use of phylogenetics (Archer et al. 2017; Taylor, Archer, et al. 2017; Nevard et al. 2020; Ferrante et al. 2022; Black et al. 2024). One generally accepted rule for demarcating subspecies is the “75% rule,” where 75% of individuals in a population are identifiable from ≥ 99% of overlapping populations (Amadon 1947; Patten and Unitt 2002; Patten 2010; Winker 2010; Taylor, Perrin, et al. 2017); however, there are multiple different methods to identify which individuals are different from overlapping populations. Utilizing multiple subspecies concepts and having a unified approach to identifying individuals that differ from overlapping populations across different subspecies concepts might lead to a more unified understanding of subspecies delineations.

North American prairie grouse (genus *Tympanuchus*) are a recently diverged group of three species of Galliformes (Greater Prairie‐Chicken [ *T. cupido* , Linnaeus, 1758], Lesser Prairie‐Chicken [ *T. pallidicinctus* , Ridgway, 1873], and Sharp‐tailed Grouse [ *T. phasianellus* , Linnaeus, 1758]) found throughout portions of the grasslands and shrublands of Canada and the United States (Galla and Johnson 2015; DeYoung and Williford 2016). This group represents a unique opportunity to study species and subspecies classifications given their recent diversification (Galla and Johnson 2015; Black et al. 2024). Sharp‐tailed Grouse are of special interest, given the recognition of six extant subspecies and one extinct subspecies (Spaulding et al. 2006; Oyler‐McCance et al. 2010; Connelly et al. 2024). The six extant subspecies of Sharp‐tailed Grouse are primarily differentiated geographically (e.g., Continental Divide and the Red River in Minnesota, North Dakota, and Manitoba), with slight differences in morphology and habitat use (Johnsgard 2016; Connelly et al. 2024). Some populations have no clear definition for their subspecies status (Spaulding et al. 2006). One population of Sharp‐tailed Grouse in south‐central Wyoming and northwestern Colorado has mixed support (genetic and morphological) for it belonging to two different subspecies of Sharp‐tailed Grouse: Columbian ( *T. phasianellus columbianus* , Ord, 1815) and plains (*T. p. jamesi*, Lincoln, 1917; Spaulding et al. 2006; Connelly et al. 2024). Columbian Sharp‐tailed Grouse are of conservation interest, given they now occupy < 10% of their historical range (Miller and Graul 1980; Hoffman et al. 2015) and they have been petitioned for listing under the Endangered Species Act of 1973 on two occasions (U.S. Department of Interior 2000, 2006). Identifying the subspecies of Sharp‐tailed Grouse for the population in south‐central Wyoming will provide a map for future identification of populations to subspecies and important information to practitioners for conservation.

The goal of our research was to apply ecological (i.e., habitat association), morphological, and genetic conceptual approaches to identify the subspecies of Sharp‐tailed Grouse inhabiting south‐central Wyoming (hereafter, unknown Sharp‐tailed Grouse). Through the use of multiple lines of evidence, we aimed to provide a robust assessment of which subspecies the unknown Sharp‐tailed Grouse population assigns to. We used ecological (i.e., habitat) characteristics, morphological variation, and genetic differentiation to test three hypotheses regarding the unknown Sharp‐tailed Grouse population: (1) the population assigns to Columbian Sharp‐tailed Grouse, (2) the population assigns to plains Sharp‐tailed Grouse, and (3) the population does not assign to either Columbian or plains Sharp‐tailed Grouse. We compared habitat characteristics, morphological characteristics, nuclear DNA microsatellite loci, and single nucleotide variants (SNVs, including insertions and deletions) derived from low‐resolution whole genome resequencing data between three groups of Sharp‐tailed Grouse, namely: known Columbian Sharp‐tailed Grouse from the nearest population in southeastern Idaho, known plains Sharp‐tailed Grouse from the nearest population in eastern Wyoming, and unknown Sharp‐tailed Grouse in south‐central Wyoming. We included Lesser Prairie‐Chicken, a closely related species within the genus *Tympanuchus*, as an outgroup to help provide discriminatory power for our analyses. Outgroups serve as a reference group for the groups that are being evaluated and are less related to the groups being evaluated than the groups being evaluated are to each other. Using Lesser Prairie‐Chicken as an outgroup allowed us to compare Sharp‐tailed Grouse populations to a related organism to better understand the differences in habitat characteristics, morphological characteristics, and single nucleotide variant data; we were not able to collect microsatellite data from Lesser Prairie‐Chickens and we did not include an outgroup in our microsatellite analyses.

## 2 Methods

**FIGURE 1:**
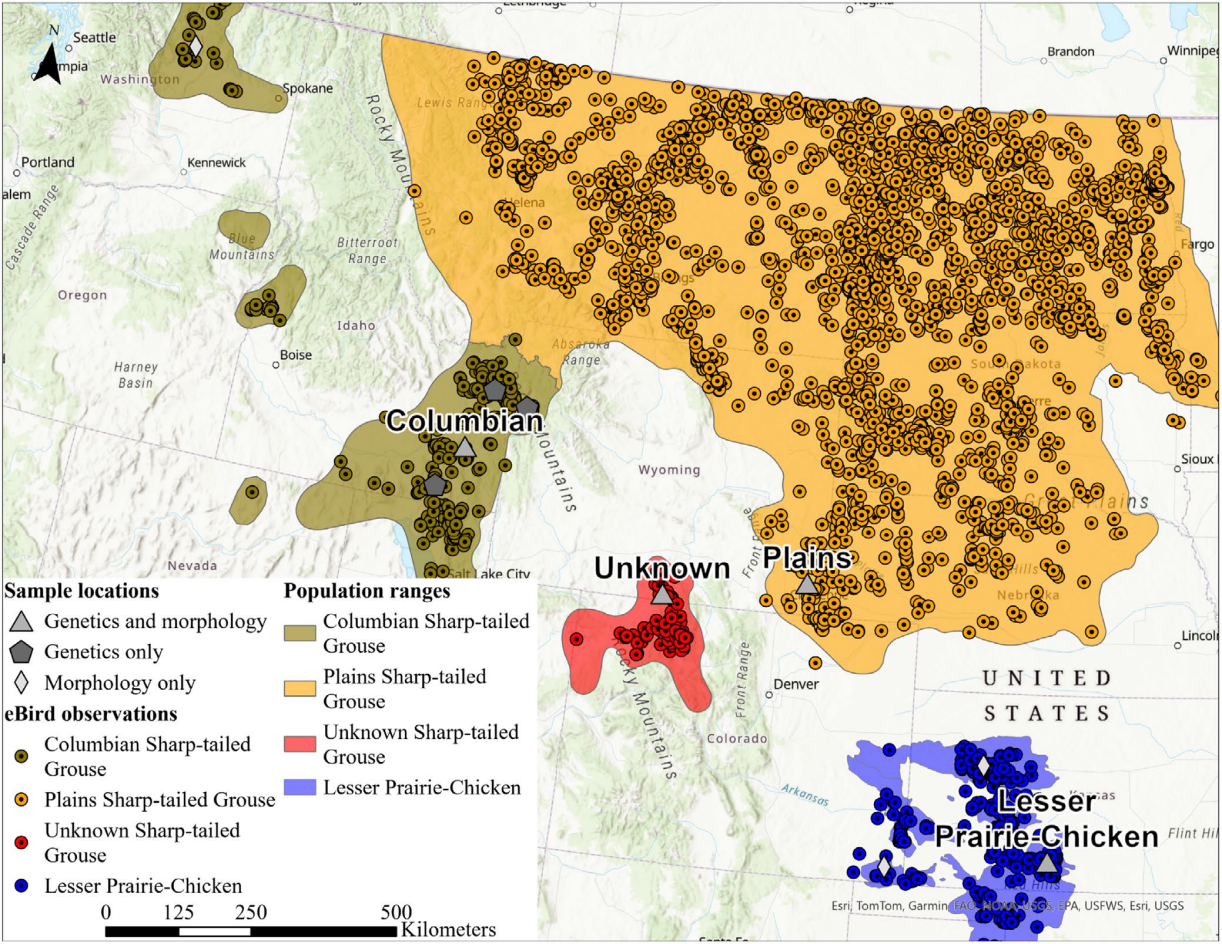
Sample locations for habitat association analysis (eBird checklist locations, 2010–2023; colored circles), morphological (gray diamonds) analysis, genetic (gray pentagons) analyses, and sites where both morphological and genetic data were collected (gray triangles). The polygons represent the estimated range for the Lesser Prairie‐Chicken (WAFWA Lesser Prairie‐Chicken Interstate Working Group 2022) and the estimated ranges for subspecies of Sharp‐tailed Grouse (Columbian, plains, and a population with an unknown subspecies) in the United States of America (Spaulding et al. 2006; Galla and Johnson 2015).

To evaluate the subspecies of Sharp‐tailed Grouse in south‐central Wyoming, we used four different datasets: habitat data, morphological data, and two genetic datasets (microsatellite genotype data and low‐resolution genome‐wide single nucleotide variants [SNVs, including insertion and deletions] data). We collected data on four populations of grouse: Lesser Prairie‐Chicken, Columbian Sharp‐tailed Grouse, plains Sharp‐tailed Grouse, and unknown Sharp‐tailed Grouse. To evaluate differences in habitat associations, we used occurrence locations from eBird, a citizen‐science database (Sullivan et al. 2009; eBird 2023). Morphological data and genetic samples for Lesser Prairie‐Chickens were collected at four different study areas in western Kansas and southeastern Colorado from 2013 to 2017; detailed descriptions of these study areas can be found in Lautenbach et al. (2019; Figure 1). We attempted to collect morphological and genetic samples from the populations of Columbian and plains Sharp‐tailed Grouse closest to our focal population in south‐central Wyoming because these are the areas that unknown Sharp‐tailed Grouse potentially interacted with in the past. Morphological data for Columbian Sharp‐tailed Grouse were collected from areas throughout Idaho and Washington in 2005–2012 (Figure 1). Genetic samples for Columbian Sharp‐tailed Grouse were collected in eastern Idaho in 2019 as well as three samples from western Wyoming in Grand Teton National Park in 2013, 2016, and 2021. Morphological and genetic data for Columbian Sharp‐tailed Grouse were collected during different years and from different locations because we were not able to collect morphological data for Columbian Sharp‐tailed Grouse; however, we were able to obtain morphological data from a previous study (Schroeder et al. 2023). We obtained genetic samples for Columbian Sharp‐tailed Grouse from hunter‐harvested wings (eastern Idaho) or road‐killed specimens (Grand Teton National Park). We collected morphological data and genetic samples for plains Sharp‐tailed Grouse in eastern Wyoming in Laramie and Goshen counties in 2019. We collected morphological data and genetic samples for unknown Sharp‐tailed Grouse in western Carbon County, Wyoming from 2017 to 2019.

### 2.1 Genetic and Morphological Field Data Collection

DNA collection techniques varied depending on the population. We captured Lesser Prairie‐Chicken, plains Sharp‐tailed Grouse, and unknown Sharp‐tailed Grouse on leks and collected blood samples from captured birds and stored these samples for later DNA extraction. We captured Lesser Prairie‐Chickens and plains and unknown Sharp‐tailed Grouse at leks using walk‐in funnel traps (Haukos et al. 1990; Schroeder and Braun 1991) and drop nets (Lesser Prairie‐Chickens only; Silvy et al. 1990). Upon capture, we collected blood via syringe from the ulnar vein or clipped the toenail of the helix toe to obtain a small sample of blood (10–30 μL). Blood samples for Lesser Prairie‐Chickens were stored in 700 μL lysis buffer (Longmire et al. 1997) and then frozen at −20°C. Blood samples for plains and unknown Sharp‐tailed Grouse were stored on Whatman FTA Micro Cards (GE Healthcare, Chicago, IL, USA) at room temperature. We collected morphological measurements from the birds we captured on leks, including culmen length (mm), head length (mm), mass (g), tail (mm), tarsus + longest toe (mm), and wing cord (mm). For Columbian Sharp‐tailed Grouse, we collected DNA samples from tissue samples collected from hunter‐harvested wings (eastern Idaho) or breast tissue from road‐killed birds (Grand Teton Nation Park, Wyoming). We obtained morphological measurements from Columbian Sharp‐tailed Grouse captured during spring for another project (Schroeder et al. 2023); morphological measurements from Columbian Sharp‐tailed Grouse included mass (g), tail (mm), tarsus + longest toe (mm), and wing cord (mm). Capturing and handling techniques for Lesser Prairie‐Chickens were approved by the Kansas State University Institutional Animal Care and Use Committee (protocol numbers 3241 and 3703), Kansas Department of Wildlife, Parks, and Tourism (scientific collection permit numbers SC‐042‐2013, SC‐079‐2014, SC‐001‐2015, and SC‐014‐2016), and Colorado Parks and Wildlife (scientific collection license numbers 13TRb2053, 14TRb2053, and 15TRb2053). Capture and handling techniques for plains and unknown Sharp‐tailed Grouse were approved by the University of Wyoming Institutional Animal Care and Use Committee (protocol 20170324AP00266 [versions −01, −02, and −03]) and by the Wyoming Game and Fish Department (Chapter 33 permits 1098 and 1214).

### 2.2 Genetic Methods

We extracted DNA from blood and muscle tissue samples using the Omega E.Z.N.A. Tissue DNA extraction kit (D3396; Omega Bio‐Tek, Norcross, GA, USA). We finely chopped up ~30 g of muscle tissue and followed the manufacturer's protocol to extract DNA. For blood samples stored on FTA micro cards, we used approximately 0.25–0.50 cm^2^ of blood on the Whatman card, finely chopped the card, and let it soak in Longmire's lysis buffer (Longmire et al. 1997) for ≥ 4 h. Following lysis, we followed the manufacturer's protocol for the remainder of the DNA extraction process.

#### 2.2.1 Microsatellite Genotyping

Once DNA was extracted, we amplified nine microsatellite loci using polymerase chain reaction (PCR). The nine microsatellite loci we amplified were ADL230 (Cheng and Crittenden 1994), BG16 (Piertney and Höglund 2001), LLSD7 (Piertney and Dallas 1997), LLST1 (Piertney and Dallas 1997), SG MS6.6 (Oyler‐McCance and St. John 2010), SG MS6.8 (Oyler‐McCance and St. John 2010), SG28 (Fike et al. 2015), TTD6 (Caizergues et al. 2001), and TUT4 (Segelbacher et al. 2000). We conducted PCR in a 12.5 μL solution, including 0.0025 nmoles forward primer, 0.0025 nmoles reverse primer, 0.0015 nmoles M13 primer, 10 ng of DNA template, and 6.25 μL of GoTaq G2 Master Mix (Promega, Madison, WI, USA). We used the published PCR amplification protocols for each primer.

#### 2.2.2 Whole Genome Resequencing

We used low resolution whole genome resequencing using single strand sequencing technologies from the third‐generation sequencing platform MinION (MinION Mk1b, Oxford Nanopore Technologies, Oxford, UK). We used the native barcoding kit SQK‐LSK109 with barcoding expansions EXP‐NBD104 and EXP‐NBD114 for ligation sequencing on genomic DNA. We followed the manufacturers' protocols for library preparation and MinION platform sequencing. We sequenced the libraries on R9.4.1 FlowCells and Flongles. We conducted base calling using *dorado* v0.3.3 within the MinKNOW software set to *super accurate* base calling. We aligned sequence reads to the Lesser Prairie‐Chicken genome (Black et al. 2023) using *minimap2* (Li 2018), indexed sequence reads using *samtools* version 1.17 (Danecek et al. 2021), and used *clair3* (Zheng et al. 2022) to call single nucleotide variants (i.e., SNVs, including insertions and deletions) at a read depth of ≥ 2X coverage. We used the *merge* tool within the *bcftools* version 1.13 (Danecek et al. 2021) to merge single nucleotide variants across all individuals and exported as a VCF file. After we merged all single nucleotide variants, we imported the VCF file into Program R version 4.4.1 (R Core Team 2024) and converted the VCF file to a GDS file using the *SNPRelate* package (Zheng et al. 2012). We then used the *snpgdsLDpruning* function within the *SNPRelate* package in Program R (Zheng et al. 2012; R Core Team 2024) to prune markers based on linkage disequilibrium, percent missingness, and minor allele frequency. We used the *composite* method built into the *snpgdsLDpruning* function and set the missing rate to 33.33%, minor allele frequency to 0.5%, a pruning window of 50 KB, and the *ld.threshold* set to 0.4 resulting in 453 single nucleotide variants used in our analyses.

### 2.3 Statistical Analysis

#### 2.3.1 General Statistical Methods

To evaluate if there were differences between our three focal populations of Sharp‐tailed Grouse and an out group for our ecological characteristics (i.e., habitat), morphological characteristics, microsatellite loci, and SNVs datasets, we conducted two main analyses: (1) discriminant analysis of principal components (DAPC; Jombart et al. 2010) and (2) a Random Forests classification model (Breiman 2001). We ran each of the described models on only our in group populations (three Sharp‐tailed Grouse populations) and all four of the populations we evaluated. The DAPC facilitated comparing different characteristics (e.g., environmental conditions, morphological measurements, microsatellite loci, or SNVs) among populations and assigning a probability of each individual bird or observation to each population based on those characteristics. We used the *xvalDapc* and *dapc* functions in the *adegenet* package in Program R (Jombart 2008; Jombart and Ahmed 2011; R Core Team 2024). Random Forests models have been used to classify subspecies using genetic data (Archer et al. 2017) but Random Forests models can also be used to classify other similar datasets. We used the *randomForest* package (Liaw and Wiener 2002) in Program R (R Core Team 2024) to run our Random Forests models. We ran Random Forests models with 10,000 trees for each forest built, and models were run with replacement. To understand the importance of variables (e.g., environmental conditions and morphological measurements) contributing to the habitat association and morphological Random Forests models, we standardized variable importance values so the top variable equaled 1, and the remaining variables were proportions derived by dividing by the top variable (Doherty et al. 2018).

#### 2.3.2 Habitat Association Analyses

To compare ecological (habitat use) differences between populations, we used DAPC and Random Forests classification models to compare environmental conditions at eBird observation locations of focal populations. To obtain eBird observation locations across the occupied ranges of Sharp‐tailed Grouse and Lesser Prairie‐Chickens in the United States, we used eBird checklists (eBird 2023) with confirmed observations for each species. We removed duplicate observations from the database prior to analyzing eBird data. For Sharp‐tailed Grouse observations, we categorized each location to a subspecies or population (Columbian Sharp‐tailed Grouse, plains Sharp‐tailed Grouse, and unknown Sharp‐tailed Grouse) based on the published ranges of each subspecies (Spaulding et al. 2006; Galla and Johnson 2015) and we used all locations for Lesser Prairie‐Chickens as our outgroup. We only used observations on checklists from January 2010 to October 2023. We filtered checklist data according to data use recommendations for using eBird data (Johnson et al. 2021; Strimas‐Mackey, Hochachka, et al. 2023); this included limiting checklists to complete checklists, checklists with distances < 5 km, < 6 h long, < 10 observers, and checklist speeds < 100 kmph (Johnson et al. 2021, Strimas‐Mackey, Hochachka, et al. 2023; see Figure 1 for map of observation locations for each population). To obtain environmental data at use locations, we used readily available remotely sensed environmental data. We obtained annual data (30‐m resolution) for annual herbaceous vegetation (biomass and cover), perennial herbaceous vegetation (biomass and cover), bare ground, litter, coniferous forest canopy cover, deciduous forest canopy cover, mixed forest canopy cover, unclassified forest canopy cover, and shrub cover from the Rangeland Analysis Platform (RAP; Robinson et al. 2019; Allred et al. 2021; Jones et al. 2021). We obtained National Land Cover Database layers (NLCD; 30‐m resolution; Jin et al. 2019) from 2011, 2013, 2016, 2019, and 2021; from each of these layers, we created multiple binary landcover layers including croplands, developed lands, emergent wetland, pasture, and water. We obtained general climate data including 30‐year average maximum temperature and 30‐year average precipitation from PRISM data (800‐m resolution; PRISM Climate Group 2014). We obtained topographic data from a 30‐m resolution digital elevation model (DEM, United States Geological Survey [USDI] 2011). From the DEM, we calculated heat load index, terrain ruggedness index, and topographic position index using the *hli*, *tri*, and *tpi* functions in the *spatialEco* package in Program R (Riley et al. 1999; McCune and Keon 2002; McCune 2007; De Reu et al. 2013; Evans and Murphy 2023; R Core Team 2024). We resampled all 30‐m grain data to 800 m using the *aggregate* and *project* functions in the package *terra* (Hijmans 2024) in Program R version 4.4.1 (R Core Team 2024) to enable comparison across all ecological covariates. Because eBird locations are not precise, we followed recommendations to use environmental variables averaged over a 1600‐m radius surrounding locations (Strimas‐Mackey, Hochachka, et al. 2023). To accommodate the imprecise locations from eBird, we used a 1600‐m moving window analysis using the *focalMat* and *focal* functions in the *terra* package (Hijmans 2024) in Program R version 4.4.1 (R Core Team 2024) to get average available conditions within 1600 m of each cell and extracted the average within each cell at each observation location. Once we extracted the environmental covariates to the eBird checklist locations, we ensured that the year for the environmental data was aligned with the year of the checklist (Jan–Dec) by aligning checklists year with year of environmental data (e.g., for checklists from Jan–Dec 2020 we used 2020 RAP). Because NLCD data was not available annually, we aligned checklists from 2010–2011 to 2011 NLCD data, checklists from 2012–2014 to 2013 NLCD data, checklists from 2015–2017 to 2016 NLCD data, checklists from 2018–2020 to 2019 NLCD data, and checklists from 2021–2023 to 2021 NLCD data. We then used a DAPC and a Random Forests model to compare environmental conditions between populations and assign a probability of each individual to each population based on habitat characteristics. To understand the differences in general habitat characteristics between populations, we used a Kruskal‐Wallis test (*α* = 0.05) and a pairwise Wilcox test (*α* = 0.05) on the six most important environmental characteristics identified in the Random Forests model.

#### 2.3.3 Morphological Analyses

To evaluate if there were differences in morphological characteristics between Columbian Sharp‐tailed Grouse, plains Sharp‐tailed Grouse, unknown Sharp‐tailed Grouse, and Lesser Prairie‐Chicken, we used a Kruskal‐Wallis test (*α* = 0.05) and a pairwise Wilcox test (*α* = 0.05) to evaluate which populations differed from each other. When evaluating morphological differences, we only used males from all populations because males and females differ in size. To evaluate if there were any differences between the three (only Sharp‐tailed Grouse) or four populations in the morphological spaces they occupied, we ran three different DAPCs models and three different Random Forests models. For both the DAPC analyses and the Random Forests models, the three models we ran were (1) an analysis on all groups including mass, (2) an analysis on all groups excluding mass, as mass can fluctuate during a season and between seasons, and (3) an analysis on only plains Sharp‐tailed Grouse, unknown Sharp‐tailed Grouse, and Lesser Prairie‐Chicken excluding mass. We included mass in one of our analyses because, generally, Columbian Sharp‐tailed Grouse are generally described as being smaller while plains Sharp‐tailed Grouse are generally described as being larger (Connelly et al. 2024) and including mass would help evaluate this. We ran the final analysis on only these three populations because we could include more morphological characteristics that were collected on all three of those populations. Specifically, for the first model including mass (all four populations), we included tail length, wing cord length, tarsus + longest toe length, mass, and all pairwise comparisons for a total of ten covariates. For the second model excluding mass (all four populations) we included tail length, wing cord length, tarsus + longest toe length, and all pairwise comparisons for a total of six covariates. For the final model (three populations: Lesser Prairie‐Chicken, plains Sharp‐tailed Grouse, and unknown Sharp‐tailed Grouse), we included wing cord length, culmen length, total head length, tarsus + longest toe length, tail length, and all pairwise combinations for a total of 15 covariates.

#### 2.3.4 Microsatellite Analyses

We scored microsatellite fragments using Geneious Prime 2022.2.2 software (https://www.geneious.com) to create an individual genetic profile for each individual. We conducted a standard assessment of microsatellite marker suitability for genetic analyses that included tests for Hardy–Weinberg Equilibrium (HWE) calculated using the *hw.test* function from the *pegas* package in Program R (Paradis 2010; R Core Team 2024), allelic richness for each population calculated using the *allel.rich* function in the *PopGenReport* package in Program R (Adamack and Gruber 2014; Gruber and Adamack 2015; R Core Team 2024), *F*_*ST*_ and *F*_*IS*_ using the *basic.stats* function from the *hierfstat* package in Program R (Goudet and Jombart 2022; R Core Team 2024), and expected heterozygosity (*H*_*E*_) and observed heterozygosity (*H*_*O*_) using the *summary* function in the *adegenet* package in Program R (Jombart 2008; Jombart and Ahmed 2011; R Core Team 2024). We checked for null alleles using the program MICRO‐CHECKER (Van Oosterhout et al. 2004) and evaluated linkage disequilibrium using the *test_LD* function in the *genepop* package in Program R (Rousset 2008; R Core Team 2024).

Following the assessments of microsatellite suitability, we used a DAPC (Jombart et al. 2010) and a Random Forests model to evaluate if there was differentiation between the populations based on microsatellite loci. We included all loci that fit the criteria that we outlined above. We only included genotype data for our in‐group populations (i.e., Sharp‐tailed Grouse, not Lesser Prairie‐Chicken).

#### 2.3.5 Whole Genome Resequencing Analyses

We calculated observed heterozygosity (*H*_*E*_), subpopulation heterozygosity (*H*_*S*_), and inbreeding coefficient (*F*_*IS*_) for each population using the *gl.basic.stats* function in the *dartR* package in Program R (Gruber et al. 2018; Mijangos et al. 2022; R Core Team 2024). We calculated pairwise proportional genetic variance (*F*_*ST*_) using the *stamppFst* function in the *StAMPP* package in Program R (Pembleton et al. 2013; R Core Team 2024). To evaluate the subspecies classification of our populations using SNVs identified above, we used a DAPC analysis and a Random Forests classification model. We used these models to compare SNVs across individuals and assign the probability of identification of each individual to each population. Once common SNVs were identified across sequenced individuals, we removed duplicate SNVs from the dataset. We obtained sequencing data from individuals in all in‐group populations (Columbian Sharp‐tailed Grouse, plains Sharp‐tailed Grouse, unknown Sharp‐tailed Grouse) and our outgroup (Lesser Prairie‐Chickens). We ran the DAPC and Random Forests model with SNVs identified to fit the criterion after pruning. For the Random Forests model, because there were missing values in the dataset, we used the *rfImpute* function from within the *randomForest* package in Program R (Liaw and Wiener 2002; R Core Team 2024) to impute missing values. We conducted the DAPC and Random Forests analyses on all of the populations and only the Sharp‐tailed Grouse populations because our sample size for Lesser Prairie‐Chickens was small (*n* = 2).

## 3 Results

We obtained 812 eBird checklist locations with Columbian Sharp‐tailed Grouse observations, 7951 eBird checklist locations with plains Sharp‐tailed Grouse observations, 509 eBird checklist locations with unknown Sharp‐tailed Grouse observations, and 1628 eBird checklist locations with Lesser Prairie‐Chicken observations (Figure S1). We obtained morphological measurements from males for 219 Columbian Sharp‐tailed Grouse, 64 plains Sharp‐tailed Grouse, 165 unknown Sharp‐tailed Grouse, and 223 Lesser Prairie‐Chicken. We obtained genotype results from nine microsatellite loci from 53 Columbian Sharp‐tailed Grouse, 32 plains Sharp‐tailed Grouse, and 175 unknown Sharp‐tailed Grouse. We identified 1750 single nucleotide variants (SNVs) with < 33.3% missing data; after pruning, we obtained 453 SNVs from 12 Columbian Sharp‐tailed Grouse, 12 plains Sharp‐tailed Grouse, 13 unknown Sharp‐tailed Grouse, and 2 Lesser Prairie‐Chickens to use for analysis. While Lesser Prairie‐chicken provided discriminatory power as our outgroup (see Appendix A), here we only present results for our in‐group populations of Sharp‐tailed Grouse.

### 3.1 Habitat Associations

Using a DAPC analysis on environmental conditions surrounding observed locations, we found that the model was able to correctly predict 65.6% of Columbian Sharp‐tailed Grouse, 98.8% of plains Sharp‐tailed Grouse, and 87.2% of unknown Sharp‐tailed Grouse (Table 1, Figure 3A). The largest proportion of the average membership probability for each population was identified as the original population of the observation (Table 2). The population with the lowest average membership probability for the population of origin was Columbian Sharp‐tailed Grouse, with an average membership probability of individuals of 65.5% Columbian Sharp‐tailed Grouse, 24.9% plains Sharp‐tailed Grouse, and 9.5% unknown Sharp‐tailed Grouse (Table 2). In general, Columbian Sharp‐tailed Grouse occupied a principal component space that overlapped both plains and unknown Sharp‐tailed Grouse; unknown and plains Sharp‐tailed Grouse did not overlap in principal component space (Figure 4A). Our Random Forests model evaluating habitat characteristics for three Sharp‐tailed Grouse populations was able to correctly classify 93.6% of Columbian Sharp‐tailed Grouse, 100% of plains Sharp‐tailed Grouse, and 98.0% of unknown Sharp‐tailed Grouse (Table 5). The three habitat characteristics with the highest importance in the Random Forests model were percent cover of shrubs (1.00), terrain ruggedness index (0.27), and heat load index (0.22; Table S1 and Figure S1).

**TABLE 1:** Number of variables, sample sizes, and assignment probabilities of discriminant analysis of principal components for populations of Columbian Sharp‐tailed Grouse (STGRc; Idaho and Washington, 2005–2013), plains Sharp‐tailed Grouse (STGRp; eastern Wyoming, 2019), and unknown Sharp‐tailed Grouse (STGRu; south‐central Wyoming, 2017–2019). Analyses were run on habitat characteristics, morphological characteristics, 9 microsatellite loci, and single nucleotide variants (SNVs).

Analyses	Number of variables	Sample size			Assignment probability		
		STGRc	STGRp	STGRu	STGRc	STGRp	STGRu
Habitat	22	812	7951	509	0.66	0.99	0.87
Morphology	6^a^	219	63	165	0.94	0.59	0.81
	10^b^	219	63	165	0.80	0.97	0.60
Microsatellite	118^c^	53	32	175	0.88	0.78	0.98
SNVs	453	12	12	13	0.92	0.83	0.92

^a^ Morphological analysis using tail length (mm), wing cord length (mm), tarsus + longest toe length (mm), and all pairwise comparisons.

^b^ Morphological analysis using mass (g), tail length (mm), wing cord length (mm), tarsus + longest toe length (mm), and all pairwise comparisons.

^c^ Total number of alleles across nine microsatellite loci.

**TABLE 2:** Mean and median average membership probability of each individual assigned to each population of prairie‐grouse evaluated using a discriminant analysis of principal components based on habitat characteristics, morphological characteristics, and single nucleotide variants for three populations of Sharp‐tailed Grouse: Columbian Sharp‐tailed Grouse (STGRc; Idaho and Washington, 2005–2013), plains Sharp‐tailed Grouse (STGRp; eastern Wyoming, 2019), and a population of Sharp‐tailed Grouse with unknown subspecific status (STGRu, south‐central Wyoming, 2017–2019) in south‐central Wyoming.

Analyses	Population	Mean (median) average membership probability of each individual		
		STGRc	STGRp	STGRu
Habitat	STGRc	0.66 (0.96)	0.25 (0.01)	0.10 (0.00)
	STGRp	0.01 (0.00)	0.99 (1.00)	0.00 (0.00)
	STGRu	0.13 (0.00)	0.00 (0.00)	0.87 (1.00)
Morphological^a^	STGRc	0.87 (0.95)	0.03 (0.01)	0.10 (0.04)
	STGRp	0.14 (0.02)	0.51 (0.52)	0.35 (0.32)
	STGRu	0.15 (0.04)	0.13 (0.06)	0.72 (0.84)
Morphological^b^	STGRc	0.69 (0.72)	0.00 (0.00)	0.31 (0.28)
	STGRp	0.01 (0.00)	0.97 (1.00)	0.02 (0.00)
	STGRu	0.43 (0.38)	0.00 (0.00)	0.57 (0.62)
Microsatellite	STGRc	0.91 (1.00)	0.02 (0.00)	0.07 (0.00)
	STGRp	0.04 (0.00)	0.79 (0.96)	0.17 (0.01)
	STGRu	0.01 (0.00)	0.02 (0.00)	0.97 (1.00)
Single nucleotide variants	STGRc	0.92 (1.00)	0.08 (0.00)	0.00 (0.00)
	STGRp	0.04 (0.00)	0.89 (1.00)	0.07 (0.00)
	STGRu	0.01 (0.00)	0.06 (0.00)	0.93 (1.00)

^a^ Morphological analysis using tail length (mm), wing cord length (mm), tarsus + longest toe length (mm), and all pairwise comparisons.

^b^ Morphological analysis using mass (g), tail length (mm), wing cord length (mm), tarsus + longest toe length (mm), and all pairwise comparisons.

### 3.2 Morphology

**FIGURE 2:**
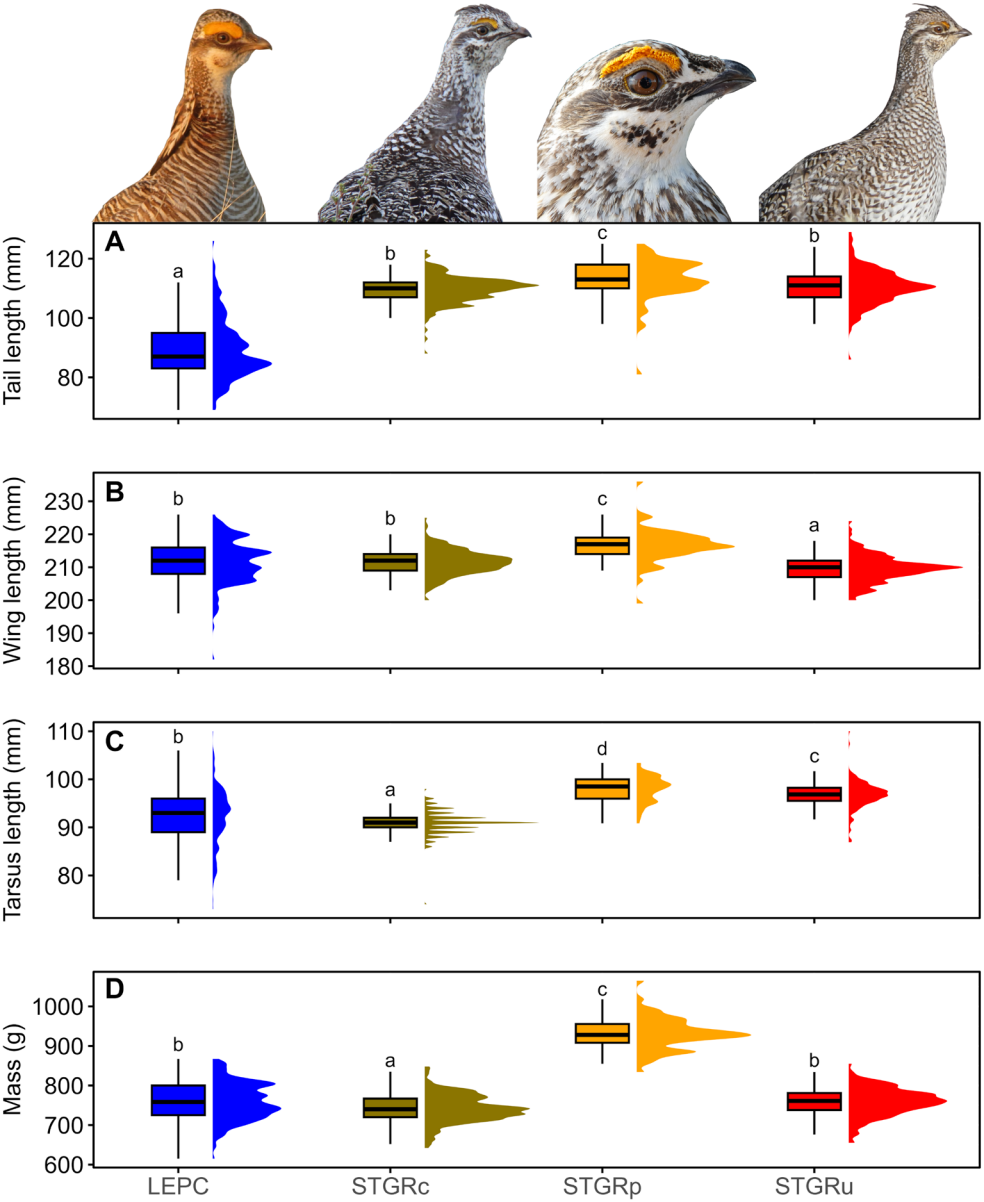
Comparison of raw morphometric measurements of males across four population of grouse: Lesser Prairie‐Chicken (LEPC; Kansas and Colorado, 2013–2017), Columbian Sharp‐tailed Grouse (STGRc; Idaho and Washington, 2005–2013), plains Sharp‐tailed Grouse (STGRp; eastern Wyoming, 2019), and a Sharp‐tailed Grouse with an unknown subspecific status (STGRu; south‐central Wyoming, 2017–2019). Morphometric measurements include tail length (mm; A), wing cord length (wing length; mm; B), tarsus + longest toe length (tarsus length; mm; C), and mass (g; D). Superscript letters above each boxplot represent statistical differences calculated using a Kruskal–Wallis rank sum test, where populations with the same letter did not differ from each other. All photos Jonathan Lautenbach.

Using morphological measurements from the three Sharp‐tailed Grouse populations, we found that there was a difference in average tail length between populations ($\chi_{2}^{2}$ = 23.37, *p* ≤ 0.001). Columbian (mean = 109.59 mm, SD = 4.55 mm) and unknown Sharp‐tailed Grouse (mean = 110.58 mm, SD = 5.92 mm) had the shortest tail lengths that did not differ from each other (*p* = 0.09) and plains Sharp‐tailed Grouse had the longest tails (mean = 112.87 mm, SD = 7.05 mm; Figure 2A). Wing cord length differed between populations ($\chi_{2}^{2}$ = 98.31, *p* ≤ 0.001). Unknown Sharp‐tailed Grouse had the shortest wing cord length (mean = 209.58 mm, SD = 4.02 mm), Columbian Sharp‐tailed Grouse (mean = 211.53 mm, SD = 3.82 mm) had intermediate wing cord lengths, and plains Sharp‐tailed Grouse had the longest wing cord lengths (mean = 216.59 mm, SD = 5.11 mm; Figure 2B). We found that tarsus + longest toe length differed between the populations ($\chi_{2}^{2}$ = 274.68, *p* ≤ 0.001). Columbian Sharp‐tailed Grouse had the shortest tarsus + longest toe length (mean = 90.95 mm, SD = 2.41 mm), unknown Sharp‐tailed Grouse (mean = 96.74 mm, SD = 2.91 mm) had intermediate tarsus + longest toe length, and plains Sharp‐tailed Grouse had the longest tarsus + longest toe length (mean = 98.00 mm, SD = 2.74 mm; Figure 2C). We found that mass differed between the three populations ($\chi_{2}^{2}$ = 180.15, *p* ≤ 0.001) with Columbian Sharp‐tailed Grouse having the lowest mass (mean = 741.80 g, SD = 35.03 g), unknown Sharp‐tailed Grouse having intermediate mass (mean = 758.92 g, SD = 34.91 g), and plains Sharp‐tailed Grouse had the greatest mass (mean = 930.05 g, SD = 40.77 g; Figure 2D).

**FIGURE 3:**
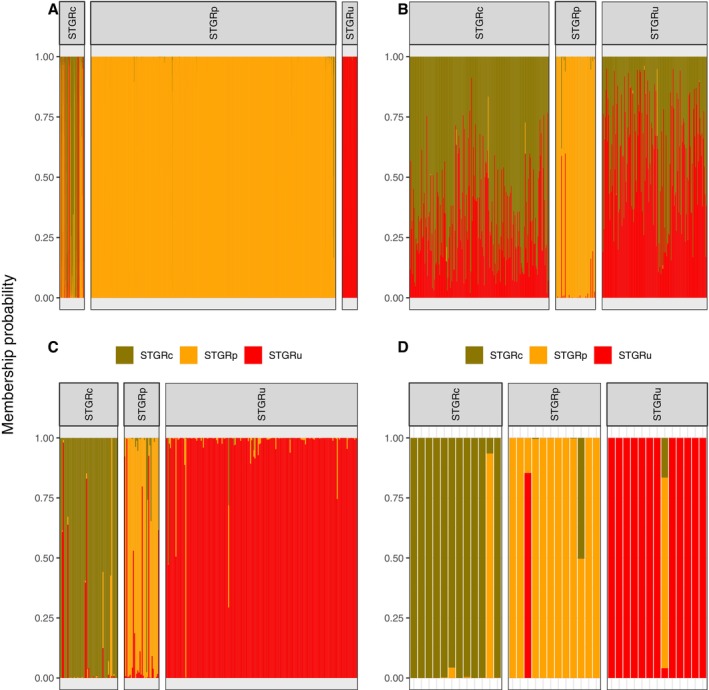
Membership probability (admixture) plots for discriminant analysis of principal components for habitat characteristics (A), morphological characteristics of males including mass (B), microsatellite loci analysis (C), and single nucleotide variants (SNVs; D) for Columbian Sharp‐tailed Grouse (STGRc; Idaho and Washington, 2005–2013, 2018), plains Sharp‐tailed Grouse (STGRp; eastern Wyoming, 2019), and unknown Sharp‐tailed Grouse subspecies (STGRu; south‐central Wyoming, 2017–2019). Membership probability plot depicts the proportion of assignment to each population, with different colors representing the proportion of each population in each individual. Facets represent the original population of each observation (habitat) or individual (morphology, microsatellite loci, and SNVs).

**FIGURE 4:**
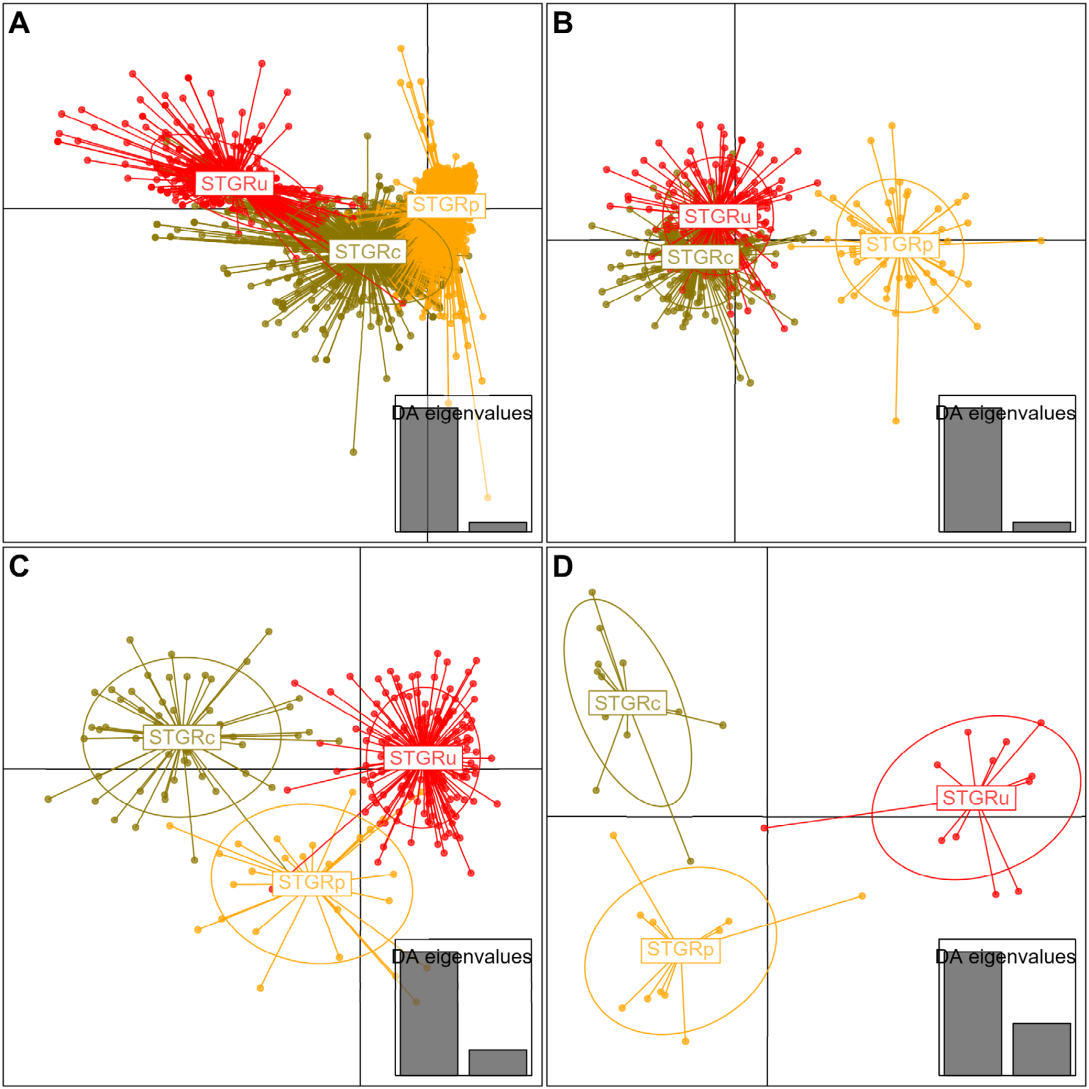
Principal component plot from discriminant analysis of principal components for habitat characteristics (A), morphological characteristics of males including mass (B), 9 microsatellite loci (C), and 453 single nucleotide variants (SNVs; D) for Columbian Sharp‐tailed Grouse (STGRc; Idaho and Washington, 2005–2013, 2018), plains Sharp‐tailed Grouse (STGRp; eastern Wyoming, 2019), and unknown Sharp‐tailed Grouse subspecies (STGRu; south‐central Wyoming, 2017–2019). Points represent individual observations (habitat) or individuals (morphology, microsatellite loci, and SNVs).

Using a discriminant analysis of principal components (DAPC) on morphological characteristics including mass on all populations, we found that this model correctly predicted the population in 79.9% of all instances for Columbian Sharp‐tailed Grouse, 96.8% of all instances for plains Sharp‐tailed Grouse, and 60.0% of all instances for unknown Sharp‐tailed Grouse (Table 1, Figure 3B). The largest proportion of the average membership probability for individuals in each population was identified as the original population of each individual (Table 2). The population with the lowest average membership probability for the population of origin was unknown Sharp‐tailed Grouse, with an average membership probability of individuals of 42.6% Columbian Sharp‐tailed Grouse, 0.2% plains Sharp‐tailed Grouse, and 57.2% unknown Sharp‐tailed Grouse (Table 2). In general, the morphological spaces of Columbian Sharp‐tailed Grouse and unknown Sharp‐tailed Grouse occupied similar spaces while plains Sharp‐tailed Grouse occupied their own principal components space (Figure 4B). Our Random Forests model evaluating three populations including mass was able to correctly classify 93.6% of Columbian Sharp‐tailed Grouse, 98.4% of plains Sharp‐tailed Grouse, and 89.1% of unknown Sharp‐tailed Grouse (Table 5). The three morphological characteristics of the highest importance in the Random Forests model were tarsus + longest toe length (1.00), wing cord length to tarsus + longest toe length ratio (0.84), and mass (0.50; Table S2).

**FIGURE 5:**
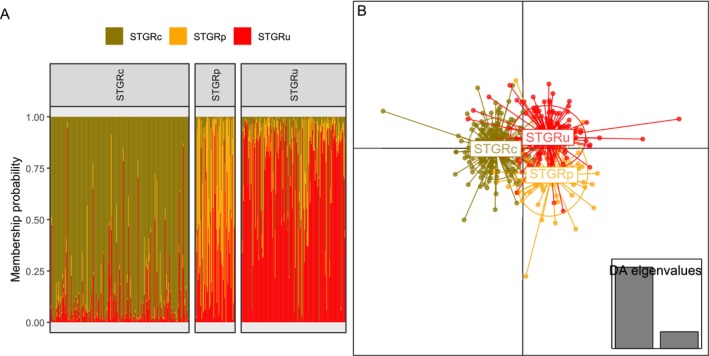
Membership probability (admixture) plot (A) and principal component plot (B) for discriminant analysis of principal components (DAPC) for three morphometric measurements of males and all pairwise comparisons for Columbian Sharp‐tailed Grouse (STGRc; Idaho and Washington, 2005–2013), plains Sharp‐tailed Grouse (STGRp; eastern Wyoming, 2019), and a Sharp‐tailed Grouse population of unknown subspecies (STGRu; south‐central Wyoming, 2017–2019).

When excluding mass from the DAPC analysis on all populations, we found that the model was able to correctly predict the population in 94.1% of all instances for Columbian Sharp‐tailed Grouse, 58.7% of all instances for plains Sharp‐tailed Grouse, and 80.6% of all instances for unknown Sharp‐tailed Grouse (Table 1, Figure 5A). The largest proportion of the average membership probability for individuals in each population was identified as the original population of each individual (Table 2). The population with the lowest average membership probability for the population of origin was plains Sharp‐tailed Grouse, with an average membership probability for individuals of 14.2% Columbian Sharp‐tailed Grouse, 50.7% plains Sharp‐tailed Grouse, and 35.0% unknown Sharp‐tailed Grouse (Table 2). In general, in this model excluding mass, plains Sharp‐tailed Grouse and unknown Sharp‐tailed Grouse occupied similar morphological spaces, while Columbian Sharp‐tailed Grouse occupied a mostly unique morphological space (Figure 5B). Our Random Forests model evaluating the three populations including mass correctly classified 91.3% of Columbian Sharp‐tailed Grouse, 58.7% of plains Sharp‐tailed Grouse, and 82.4% of unknown Sharp‐tailed Grouse (Table 5). The three morphological characteristics of the highest importance in the Random Forests model were tarsus + longest toe length (1.00), the wing cord length to tarsus + longest toe length ratio (0.78), and wing cord length (0.40; Table S3).

### 3.3 Microsatellite Genotyping

The number of alleles at each loci varied (7–23; Table 3). Allelic richness in each population varied, with the highest average allelic richness occurring in the plains Sharp‐tailed Grouse population (mean = 8.43), followed by Columbian Sharp‐tailed Grouse (mean = 8.13), and unknown Sharp‐tailed Grouse had the average lowest allelic richness (mean = 7.02; Table 3). Hardy–Weinberg exact tests indicated significant deviations from Hardy–Weinberg equilibrium expectations for several markers (Table 3). Deviations from Hardy–Weinberg equilibrium were expected given that our dataset includes individuals from multiple subspecies and populations that were separated by significant distances. Tests for linkage disequilibrium indicated linkage between several markers; however, these markers were only significant in one population. In our Columbian Sharp‐tailed Grouse population, ADL230 and LLSD7 showed linkage (*p* = 0.035) and ADL230 and SGMS06.8 showed linkage (*p* = 0.006). For unknown Sharp‐tailed Grouse, LLST1 and TUT4 showed linkage (*p* = 0.017). The markers LLST1 and TUT4 showed weak evidence of having null alleles present due to excess homozygotes for most allele classes, as indicated by MICRO‐CHECKER in our unknown Sharp‐tailed Grouse population, likely due to latent population structure and likely not to influence our results (Van Oosterhout et al. 2006). In exploratory analyses removing either LLST1 or TUT4, our results did not change.

**TABLE 3:** Population genetic summary statistics for 9 microsatellite loci from 53 Columbian Sharp‐tailed Grouse (STGRc, Idaho, 2018), 32 plains Sharp‐tailed Grouse (STGRp, eastern Wyoming, 2019), and 175 unknown Sharp‐tailed Grouse (STGRu, south‐central Wyoming, 2017–2019). We report the number of alleles (*N*_*A*_), allelic richness (*AR*) by populations, inbreeding coefficient (*F*_*IS*_), proportional genetic variance (*F*_*ST*_), expected heterozygosity (*H*_*E*_), observed heterozygosity (*H*_*O*_), and Hardy–Weinberg Equilibrium *p*‐value (*HWE*).

Loci	*N*_*A*_	*AR*_*STGRc*_	*AR*_*STGRp*_	*AR*_*STGRu*_	*F*_*IS*_	*F*_*ST*_	*H*_*O*_	*H*_*E*_	*HWE*
ADL230	11	7.74	8.98	3.63	0.00	0.08	0.63	0.66	< 0.001
BG16	11	7.48	7.62	7.65	−0.03	0.06	0.81	0.82	0.107
LLSD7	13	8.62	9.85	8.78	−0.04	0.01	0.85	0.81	0.022
LLST1	7	5.69	4.00	4.11	0.21	0.06	0.45	0.66	< 0.001
SGMS06.6	23	13.00	13.52	12.42	−0.02	0.03	0.87	0.87	< 0.001
SGMS06.8	11	7.51	8.23	5.58	−0.06	0.03	0.81	0.75	0.247
SG28	17	7.03	5.95	3.98	−0.02	0.02	0.22	0.22	1.000
TUT4	15	10.17	9.99	10.95	0.13	0.03	0.67	0.89	< 0.001
TTD6	10	5.97	7.75	6.09	0.07	0.06	0.59	0.67	< 0.001

Using a DAPC analysis on genotype data from microsatellites, we found that the model was able to correctly predict the population 90.5% of the time for Columbian Sharp‐tailed Grouse, 78.1% of the time for plains Sharp‐tailed Grouse, and 98.3% of the time for unknown Sharp‐tailed Grouse (Table 1; Figure 3C). The largest proportion of the average membership probability for individuals in each population was identified as the original population of each individual (Table 2). The population with the lowest average membership probability for the population of origin was plains Sharp‐tailed Grouse, with an average membership probability for individuals of 5.5% Columbian Sharp‐tailed Grouse, 77.6% plains Sharp‐tailed Grouse, and 16.9% unknown Sharp‐tailed Grouse (Table 2). In general, the principal components space of Columbian Sharp‐tailed Grouse, plains Sharp‐tailed Grouse, and unknown Sharp‐tailed Grouse were unique, with each species occupying its own spaces (Figure 4C). Our Random Forests model evaluating 9 microsatellites across all individuals genotyped was able to correctly classify 75.5% of Columbian Sharp‐tailed Grouse, 31.3% of plains Sharp‐tailed Grouse, and 98.3% of unknown Sharp‐tailed Grouse (Table 5).

### 3.4 Whole Genome Resequencing

Diversity (*H*_*O*_, *H*_*S*_, *F*_*IS*_) across our in‐group (Columbian Sharp‐tailed Grouse, plains Sharp‐tailed Grouse, and unknown Sharp‐tailed Grouse) populations was low, but consistent across populations, with our out‐group (Lesser Prairie‐Chicken) showing lower diversity (Table 4). Pairwise genetic differentiation (*F*_*ST*_) was low among our in‐group populations, with greater differentiation between our in‐group population and our out‐group population (Table 4). Using a DAPC on low‐resolution whole genome resequencing single nucleotide variant data, the model was able to correctly predict 91.7% of Columbian Sharp‐tailed Grouse, 83.3% of plains Sharp‐tailed Grouse, and 92.3% of unknown Sharp‐tailed Grouse (Table 1, Figure 3D). All populations had a high proportion of the average membership probability (> 85.0%) for individuals in each population identified as the original population of each individual (Table 2). In general, all three populations of Sharp‐tailed Grouse occupied a unique principal component space (Figure 4D). Our Random Forests model evaluating 453 SNVs across all Sharp‐tailed Grouse populations was able to correctly classify 41.7% of Columbian Sharp‐tailed Grouse, 41.7% of plains Sharp‐tailed Grouse, and 46.2% of unknown Sharp‐tailed Grouse (Table 5).

**TABLE 4:** Sample size (*n*), proportional genetic variance (*F*_*ST*_), observed heterozygosity (*H*_*O*_), subpopulation heterozygosity (*H*_*S*_), and inbreeding coefficient (*F*_*IS*_) for 453 single nucleotide polymorphisms and insertions and deletions loci from 39 *Tympanuchus* samples (Lesser Prairie‐Chicken [LEPC; Kansas, 2013], Columbian Sharp‐tailed Grouse [STGRc; Idaho, 2018], plains Sharp‐tailed Grouse [STGRp; eastern Wyoming, 2019], and unknown Sharp‐tailed Grouse [STGRu; south‐central Wyoming, 2017–2019]).

Subspecies	*n*	*F*_*ST*_ LEPC	*F*_*ST*_ STGRc	*F*_*ST*_ STGRp	*H*_*O*_	*H*_*S*_	*F*_*IS*_
LEPC	2	—	—	—	0.19	0.11	−0.74
STGRc	12	0.06	—	—	0.22	0.16	−0.39
STGRp	12	0.00	0.02	—	0.22	0.17	−0.35
STGRu	13	0.16	0.06	0.04	0.23	0.17	−0.38

**TABLE 5:** Pairwise comparisons and classification error of Random Forests classification for three populations of Sharp‐tailed Grouse based on habitat characteristics, morphological characteristics, microsatellite loci, and single nucleotide variants (SNVs). Sharp‐tailed Grouse populations evaluated are Columbian Sharp‐tailed Grouse (STGRc; Idaho and Washington, 2005–2013, 2018), plains Sharp‐tailed Grouse (STGRp; eastern Wyoming, 2019), and unknown Sharp‐tailed Grouse (STGRu; south‐central Wyoming, 2017–2019).

Analysis	Population	STGRc	STGRp	STGRu	Classification error (%)
Habitat	STGRc	760	51	1	6.4
	STGRp	4	7947	0	0.1
	STGRu	9	1	499	2.0
Morphological^a^	STGRc	200	4	15	8.7
	STGRp	6	37	20	41.3
	STGRu	16	13	136	17.6
Morphological^b^	STGRc	205	1	13	6.4
	STGRp	0	62	1	1.6
	STGRu	17	1	147	10.9
Microsatellite loci	STGRc	40	2	11	24.5
	STGRp	3	10	19	68.8
	STGRu	2	1	172	1.7
SNVs	STGRc	6	2	4	58.3
	STGRp	3	5	4	58.3
	STGRu	4	2	7	46.2

^a^ Morphological analysis using tail length (mm), wing cord length (mm), tarsus + longest toe length (mm), and all pairwise comparisons.

^b^ Morphological analysis using mass (g), tail length (mm), wing cord length (mm), tarsus + longest toe length (mm), and all pairwise comparisons.

## 4 Discussion

Historically, Sharp‐tailed Grouse subspecies have been demarcated using geographic boundaries (e.g., Continental Divide or the Red River in Minnesota, North Dakota, and Manitoba), differences in habitat, and slight differences in morphology and plumage (Aldrich and Duvall 1955; Connelly et al. 2024). We evaluated the subspecific status of a population of Sharp‐tailed Grouse in south‐central Wyoming using habitat, morphological, and genetic (microsatellite and genome‐wide SNVs) characteristics. Our results suggest that the three populations of Sharp‐tailed Grouse that we evaluated form three unique groups. This pattern is most evident when using habitat associations, microsatellite markers, and single nucleotide variants across the three Sharp‐tailed Grouse populations. Differences between populations were less pronounced when evaluating morphological characteristics; however, there were still differences between the populations when both including and excluding mass in the comparisons. Including Lesser Prairie‐Chicken as an outgroup in our analysis (see Appendix A for results including outgroup) provided a strong discriminatory power and showed similar results, with our three populations of Sharp‐tailed Grouse generally forming their own groups and Lesser Prairie‐Chicken forming its own group as well. Overall, our results suggest that the Sharp‐tailed Grouse population in south‐central Wyoming is different from both the plains and Columbian Sharp‐tailed Grouse subspecies and might represent a different subspecies.

Typically, subspecies have been described using a single approach, ranging from ecological differences (Philips 1948; Wilson et al. 2010; Khimoun et al. 2013), morphological differences (Pivnička 1970; Owen and Webster 1983; Marantz and Patten 2010), and genetic differences (Funk et al. 2007; Archer et al. 2017; Ferrante et al. 2022). While many studies use a single approach, there are some that use multiple approaches to evaluate subspecies (e.g., Zink 2015; Meiri et al. 2017; Walsh et al. 2017). We used multiple data types (e.g., habitat, morphology, and genetic) and analytical approaches (e.g., DAPC and Random Forests) to evaluate the Sharp‐tailed Grouse population in south‐central Wyoming, with the results from the different data types and analytical approaches generally aligning. However, there were some discrepancies depending on which analytical approach was used. The two analytical approaches (DAPC and Random Forests models) for morphological, microsatellite, and SNVs did not always agree, with the Random Forests models typically having poorer performances than DAPC models. Specifically, the Random Forests classification models had poorer performances when we used smaller sample sizes or included fewer variables in the analysis, and the poorer performance is likely a result of not having adequate sample sizes or including enough predictor variables (Archer et al. 2017; Brieuc et al. 2018; Luan et al. 2020). For example, the Random Forests model evaluating SNVs performed poorly, and we only had 12–13 samples per population. Conversely, with more information to discern differences among sample groups, our morphological Random Forest analysis was able to better discriminate among the three populations when we included mass as a covariate (note, all variables that included mass had intermediate importance in the Random Forest model; Table S2) had lower classification error than when we excluded mass from the model.

The majority of the DAPC models correctly differentiated and identified the population of origin of > 75% of all the individuals evaluated, except for morphology characteristics and habitat associations models. Morphological differences between the populations were less pronounced, and differentiation depended on what variables were included in the analysis. When mass was included in the analysis, unknown Sharp‐tailed Grouse were similar to Columbian Sharp‐tailed Grouse; however, when mass was excluded from the analysis, unknown Sharp‐tailed Grouse were similar to plains Sharp‐tailed Grouse. Further, when we included additional morphological measurements, unknown Sharp‐tailed Grouse were different from plains Sharp‐tailed Grouse (see Appendix A for results). This suggests that the number of morphological measurements is important, and a study that includes more morphological characteristics for all populations will provide better insight into morphological differentiation. For the habitat associations model, the DAPC was able to correctly differentiate habitat use for the unknown subspecific population in south‐central Wyoming from Columbian and plains Sharp‐tailed Grouse habitat; however, it did a poor job of differentiating Columbian Sharp‐tailed Grouse from plains Sharp‐tailed Grouse habitat. The inability of the DAPC model to differentiate Columbian Sharp‐tailed Grouse habitat characteristics from plains Sharp‐tailed Grouse habitat characteristics likely stems from Columbian Sharp‐tailed Grouse occupying Conservation Reserve Program grasslands in portions of their range (Hoffman et al. 2015; Stevens et al. 2023); Conservation Reserve Program grasslands found within the Columbian Sharp‐tailed Grouse range likely share characteristics with grasslands found throughout much of the plains Sharp‐tailed Grouse range.

Across their range, Sharp‐tailed Grouse populations inhabit a variety of different habitats ranging from grasslands with no shrubs or trees to open clearings surrounded by predominately closed canopy forests, with occupied habitats generally differing among subspecies (Johnsgard 2016; Connelly et al. 2024). Our results help confirm that there are differences in habitat use between some populations of Sharp‐tailed Grouse, with Sharp‐tailed Grouse in south‐central Wyoming generally occupying different habitat conditions than Columbian and plains Sharp‐tailed grouse. The habitats that Sharp‐tailed Grouse typically inhabit do not include continuous conifer forests, alpine areas, or high deserts, which are the predominant habitats found surrounding the area occupied by the unknown Sharp‐tailed Grouse population. This large expanse of uninhabited areas surrounding the unknown Sharp‐tailed Grouse population has resulted in the population being isolated from other populations of Sharp‐tailed Grouse (Columbian and plains subspecies). Evidence suggests that *Tympanuchus* grouse can disperse over large distances, with potential dispersal distances up to 120 km in some areas (Earl et al. 2016; Roy and Gregory 2019), with these dispersal events occurring in human‐fragmented landscapes with patches of suitable habitat between them. The ranges of both the Columbian and plains Sharp‐tailed Grouse are located farther from the maximum distance a *Tympanuchus* grouse has been observed to disperse (175 and 130 km, respectively) with most of the distance between them representing unsuitable habitat, though a potential dispersal event cannot be ruled out. The isolation of the unknown Sharp‐tailed Grouse population was likely not human‐caused, unlike the fragmentation of Columbian Sharp‐tailed Grouse in Idaho, Utah, and Oregon (Miller and Graul 1980; Hoffman et al. 2015). Additional research is needed to evaluate the timeframe that the unknown Sharp‐tailed Grouse population was isolated from other Sharp‐tailed Grouse populations.

In our study, we used two different genetic approaches to discriminate populations: microsatellite loci and single nucleotide variants generated from low resolution whole genome resequencing. Results from both datasets analyzed using a DAPC indicated that the three populations of Sharp‐tailed Grouse were different from each other, with few differences in discriminatory power between the two approaches. In other non‐model systems, SNVs have shown more resolution to discriminate among populations than microsatellites, especially when populations are genetically depauperate (Galla et al. 2020; Zimmerman et al. 2020; Hauser et al. 2021; but see also Forsdick et al. 2021); this power often comes from the quantity of data obtained from high throughput sequencing approaches. We did not see strong differences between microsatellites and SNVs here, and we have considered two explanations for this pattern. First, the SNVs called here were produced at 2× read depth using low resolution resequencing Nanopore data on relatively few individuals, which may have resulted in fewer SNVs and discriminatory power compared to microsatellites. Second, the microsatellite dataset here is robust, with a high number of possible alleles per locus, which may have allowed for substantial discriminatory power. We contend that both approaches were capable of discriminating populations in our analyses and therefore may be useful to others exploring this approach.

Our results indicate that the population of Sharp‐tailed Grouse in south‐central Wyoming differs from Columbian and plains Sharp‐tailed Grouse using ecological, morphological, and genetic approaches. To fully evaluate the subspecific status, analyses including evolutionary relationships will provide a better understanding of which populations of Sharp‐tailed Grouse belong to which subspecies. A recent phylogenetic study indicated high nodal support for branching between many Sharp‐tailed Grouse subspecies, including plains and Columbian, depending on the genomic markers used (e.g., autosomal, Z‐linked, intergenic, and genic sites; Johnson et al. 2023). While the sample size was small for Sharp‐tailed Grouse from south‐central Wyoming (*n* = 2), there is support for differentiation from plains and Columbian Sharp‐tailed Grouse in some—but not all—species trees. This complexity may be due to the recent evolutionary history of North American prairie grouse (i.e., incomplete lineage sorting), hybridization between taxa, and pre‐zygotic barriers of sexual selection (Galla and Johnson 2015). We recommend a more targeted phylogenomic study across Sharp‐tailed Grouse, including adding samples from northwest Colorado (which is connected to the population in south‐central Wyoming) and increased sample sizes for plains and Columbian Sharp‐tailed Grouse from across their distributions to elucidate this complicated history. Finally, it should be noted that there was a historic population of Sharp‐tailed Grouse in New Mexico that was classified as a separate subspecies ( *Tympanuchus phasianellus hueyi* ; Dickerman and Hubbard 1994). The plumage description of these birds by Dickerman and Hubbard (1994) is similar to what we observed in the population in south‐central Wyoming (Lautenbach and Pratt, personal observation). The evolutionary history of this population remains unknown and could be assessed in relationship to historic and contemporary Sharp‐tailed Grouse populations in the western United States, using museum specimens.

### 4.1 Conservation Implications

Our results may potentially change the current understanding of Sharp‐tailed Grouse subspecies in western North America, which can impact how to manage them. Our results have particularly important implications for Columbian Sharp‐tailed Grouse, a subspecies that has been petitioned for listing under the Endangered Species Act (U.S. Department of Interior 2000, 2006). Historically, the population in south‐central Wyoming extending into northwestern Colorado was thought to be the Columbian subspecies. Excluding the estimated population size of Sharp‐tailed Grouse in northwest Colorado and south‐central Wyoming (approximately 8000–10,000 birds; Hoffman 2001; Mong et al. 2017) reduces the estimated population of Columbian Sharp‐tailed Grouse by about 10%–20% (Columbian Sharp‐tailed Grouse population is estimated to be between 41,000–62,000; Gillette 2014; Chutter 2015; Schroeder et al. 2023). Not only do our results potentially change our understanding of population sizes, but they also could change how we manage these populations, including habitat management and translocations. To maintain the genetic integrity of Columbian Sharp‐tailed Grouse populations, our results indicate managers should not use the population in south‐central Wyoming (and likely northwest Colorado) as a source population for reintroduction and population augmentation efforts of Columbian Sharp‐tailed Grouse in places with small or extirpated populations (e.g., Nevada, Oregon, and Washington). Our analyses of habitat conditions suggest that there are some habitat differences between south‐central Wyoming/northwestern Colorado and Columbian Sharp‐tailed Grouse populations. Currently, habitat management actions are applied uniformly between Columbian Sharp‐tailed Grouse and populations of Sharp‐tailed grouse in south‐central Wyoming and northwest Colorado (Hoffman et al. 2015). Our results suggest a need to reevaluate habitat management approaches for Sharp‐tailed Grouse across the range of these species/subspecies in Wyoming, Idaho, and northwest Colorado.

## Author Contributions

**Jonathan D. Lautenbach:** conceptualization (equal), data curation (lead), formal analysis (lead), funding acquisition (supporting), investigation (equal), methodology (equal), visualization (lead), writing – original draft (lead), writing – review and editing (equal). **Andrew J. Gregory:** conceptualization (equal), data curation (equal), funding acquisition (supporting), investigation (supporting), methodology (equal), project administration (supporting), supervision (supporting), writing – original draft (supporting), writing – review and editing (equal). **Stephanie Galla:** methodology (supporting), writing – original draft (supporting), writing – review and editing (equal). **Aaron C. Pratt:** conceptualization (equal), funding acquisition (supporting), investigation (supporting), methodology (supporting), project administration (supporting), supervision (supporting), writing – review and editing (equal). **Michael A. Schroeder:** data curation (supporting), funding acquisition (supporting), writing – review and editing (equal). **Jeffrey L. Beck:** conceptualization (equal), data curation (supporting), funding acquisition (lead), investigation (equal), methodology (supporting), project administration (lead), supervision (lead), writing – original draft (supporting), writing – review and editing (equal).

## Conflicts of Interest

The authors declare no conflicts of interest.

## Supporting information


Appendix A.

Table S1.
Table S2.
Table S3.
Figure S1.

## Data Availability

Habitat association, morphology, microsatellite, and single nucleotide variant data are available on Dryad (https://doi.org/10.5061/dryad.gf1vhhmzz). Raw eBird observations and records are archived and freely and publicly accessible through eBird. Spatial data used in the habitat association analyses are available for download from the source (see Methods for where these data were obtained from). All code (R code and Linux code) used to generate, process, and analyze data are available on Dryad (https://doi.org/10.5061/dryad.gf1vhhmzz). Raw sequence reads and sequence sample metadata are deposited in the SRA (BioProject PRJNA1196947).
