# Supplementary material for: Using Habitat, Morphological, and Genetic Characteristics to Delineate the Subspecies of Sharp‐Tailed Grouse in South‐Central Wyoming
Source: Ecol Evol. 2025 May 12;15(5):e71429. doi: 10.1002/ece3.71429 (PMC12068902; doi:10.1002/ece3.71429)
Supplement: Supplementary file 1 — Appendix A. [file ECE3-15-e71429-s001.docx]

**APPENDIX A:**

Appendix A reports results for the discriminatory analysis including Lesser Prairie-Chicken (*Tympanuchus pallidicinctus*) as an outgroup when evaluating the relationship of three populations of Sharp-tailed Grouse: Columbian Sharp-tailed Grouse (*T. phasianellus columbianus*), plains Sharp-tailed Grouse (*T. phasianellus jamesi*), and a population of unknown subspecific status in south-central Wyoming (hereafter unknown Sharp-tailed Grouse). Please see the Methods section in the main manuscript for a description of the methods used. Note, we do not include results for microsatellite analyses in this Appendix because we did not collect data on microsatellite loci for Lesser Prairie-Chickens.

**RESULTS**

**Habitat**—A discriminant analysis of principal components (DAPC) on habitat conditions surrounding observed locations correctly predicted 100.0% of Lesser Prairie-Chicken observations, 66.0% of Columbian Sharp-tailed Grouse observations, 97.8% of plains Sharp-tailed Grouse observations, and 83.7% of unknown Sharp-tailed Grouse observations (Table 1, Figure A1c). Average membership probabilities for Lesser Prairie-Chicken, plains Sharp-tailed Grouse, and unknown Sharp-tailed Grouse observations were relatively high (1.00, 0.98, and 0.85, respectively) while Columbian Sharp-tailed Grouse were intermediate (0.66; Table A2). Average membership probabilities for Columbian Sharp-tailed Grouse were 0.25 plains Sharp-tailed Grouse and 0.09 unknown Sharp-tailed Grouse (Table A2). In general, Lesser Prairie-Chickens occupied their own principal components space while the principal components space of Columbian Sharp-tailed Grouse overlapped both plains and unknown Sharp-tailed Grouse; unknown and plains Sharp-tailed Grouse did not overlap in principal components space (Figure A4d). Our Random Forests model evaluating habitat conditions for Lesser Prairie-Chickens and three Sharp-tailed Grouse populations correctly classified 100% of Lesser Prairie-Chickens, 93.3% of Columbian Sharp-tailed Grouse, 100% of plains Sharp-tailed Grouse, and 98.4% of unknown Sharp-tailed Grouse (Table A5). The three habitat characteristics with the highest importance in the Random Forests model were mean annual maximum temperature (1.00), shrub cover (0.55), and precipitation (0.21; Table A4).

When evaluating the species distribution models for the populations, we observed a similar relationship to the DAPC analysis, with Columbian Sharp-tailed Grouse habitat occurring in portions of unknown and plains Sharp-tailed Grouse with no overlap of habitat between plains Sharp-tailed Grouse, unknown Sharp-tailed Grouse, and Lesser Prairie-Chickens (Figure 1).

**Morphology**—Using morphological measurements from the four populations, we found that there was a difference in average tail length between populations ($\chi_{3}^{2}$= 372.26, *P* ≤ 0.001), with Lesser Prairie-Chickens having the shortest tail (mean = 89.28 mm, SD = 9.90 mm), Columbian (mean = 109.59, SD = 4.55 mm) and unknown Sharp-tailed Grouse (mean = 110.58 mm, SD = 5.92 mm) had intermediate tail lengths that did not differ from each other (*P* = 0.09), and plains Sharp-tailed Grouse had the longest tails (mean = 112.87 mm, SD = 7.05 mm; Figure 2A). Wing cord length differed between populations ($\chi_{3}^{2}$= 88.85, *P* ≤ 0.001) with unknown Sharp-tailed Grouse having the shortest wing cord (mean = 209.58 mm, SD = 4.02 mm), Lesser Prairie-Chicken (mean = 211.89 mm, SD = 6.52) and Columbian Sharp-tailed Grouse (mean = 211.53 mm, SD = 3.82 mm) having intermediate wing cord lengths that did not differ from each other (*P* = 0.32), and plains Sharp-tailed Grouse had the longest wing cord lengths (mean = 216.59 mm, SD = 5.11 mm; Figure 2B). We found that tarsus + longest toe length differed between the populations ($\chi_{3}^{2}$= 88.85, *P* ≤ 0.001) with Columbian Sharp-tailed Grouse having the shortest tarsus + longest toe length (mean = 90.95 mm, SD = 2.41 mm) followed by Lesser Prairie-Chickens (mean = 92.30 mm, SD = 5.95), unknown Sharp-tailed Grouse (mean = 96.74, SD = 2.91 mm), and plains Sharp-tailed Grouse had the longest tarsus + longest toe length (mean = 98.00 mm, SD = 2.74 mm; Figure 2C). We found that mass differed between the four populations ($\chi_{3}^{2}$= 194.57, *P* ≤ 0.001) with Columbian Sharp-tailed Grouse having the lowest mass (mean = 741.80 g, SD = 35.03 g), Lesser Prairie-Chicken (mean = 760.02 g, 46.55 g) and unknown Sharp-tailed Grouse (mean = 758.92 g, SD = 34.91 g) having intermediate mass that did not differ from each other (*P* = 0.98), and plains Sharp-tailed Grouse had the greatest mass (mean = 930.05 g, SD = 40.77 g; Figure 2D).

Using a discriminant analysis of principal components (DAPC) on morphological characteristics including mass on all populations, we found that this model correctly predicted the population 84.2% of the time for Lesser Prairie-Chickens, 93.2% of the time for Columbian Sharp-tailed Grouse, 96.8% of the time for plains Sharp-tailed Grouse, and 81.8% of the time for unknown Sharp-tailed Grouse (Table A1, Figure A1a). Average membership probabilities for Lesser Prairie-Chicken and plains Sharp-tailed Grouse individuals were relatively high (0.81 and 0.96, respectively), while average membership probabilities for Columbian Sharp-tailed Grouse and unknown Sharp-tailed Grouse individuals were intermediate (0.75 and 0.65; Table A2). Average membership probabilities for Columbian Sharp-tailed Grouse were 0.04 Lesser Prairie-Chicken and 0.21 unknown Sharp-tailed Grouse (Table A2). Average membership probabilities for unknown Sharp-tailed Grouse were 0.04 Lesser Prairie-Chicken and 0.30 Columbian Sharp-tailed Grouse (Table A2). In general, the morphological spaces of Columbian Sharp-tailed Grouse and unknown Sharp-tailed Grouse occupied similar spaces while Lesser Prairie-Chickens and plains Sharp-tailed Grouse occupied their own spaces (Figure A2a). Our Random Forests model evaluating four populations including mass correctly classified 87.4% of Lesser Prairie-Chickens, 88.1% of Columbian Sharp-tailed Grouse, 95.2% of plains Sharp-tailed Grouse, and 84.9% unknown Sharp-tailed Grouse (Table A3). The three morphological factors with the highest importance in the Random Forests model when including mass were the wing cord length to tail length ratio (1.00), tarsus + longest toe length (0.91), and wing cord length to tarsus + longest toe length ratio (0.76; Table A5).

When excluding mass from the DAPC analysis on all populations, we found that this model correctly predicted the population 83.9% of the time for Lesser Prairie-Chickens, 94.5% of the time for Columbian Sharp-tailed Grouse, 41.3% of the time for plains Sharp-tailed Grouse, and 77.0 % of the time for unknown Sharp-tailed Grouse (Table A1, Figure A3a). Average membership probabilities for Lesser Prairie-Chicken individuals were relatively high (0.80), while average membership probabilities for Columbian Sharp-tailed Grouse, plains Sharp-tailed Grouse, and unknown Sharp-tailed Grouse individuals were intermediate to low (0.74, 0.40, and 0.59, respectively; Table A2). Average membership probabilities for Columbian Sharp-tailed Grouse were 0.03 Lesser Prairie-Chicken, 0.05 plains Sharp-tailed Grouse, and 0.18 unknown Sharp-tailed Grouse (Table A2). Average membership probabilities for plains Sharp-tailed Grouse were 0.05 Lesser Prairie-Chicken, 0.19 Columbian Sharp-tailed Grouse, and 0.36 unknown Sharp-tailed Grouse (Table A2). Average membership probabilities for unknown Sharp-tailed Grouse were 0.04 Lesser Prairie-Chicken, 0.25 Columbian Sharp-tailed Grouse, and 0.12 plains Sharp-tailed Grouse (Table A2). In general, in this model excluding mass, plains Sharp-tailed Grouse and unknown Sharp-tailed Grouse occupied similar morphological spaces (Figure A3b), while Columbian Sharp-tailed Grouse occupied a mostly unique morphological spaces, and Lesser Prairie-Chickens occupied their own space (Figure A3a). Our Random Forests model evaluating four populations while excluding mass correctly classified 87.4% of Lesser Prairie-Chickens, 88.1% of Columbian Sharp-tailed Grouse, 52.4% of plains Sharp-tailed Grouse, and 76.4% of unknown Sharp-tailed Grouse (Table A3). The three morphological factors with the highest importance in the wing cord length to tail length ratio (1.00), tarsus + longest toe length (0.93), and tail length (0.74; Table A6).

When evaluating morphological differences between Lesser Prairie-Chicken, plains Sharp-tailed Grouse, and unknown Sharp-tailed Grouse using 15 covariates, we found that the model correctly predicted the population 92.8% of the time for Lesser Prairie-Chickens, 71.4% of the time for plains Sharp-tailed Grouse, and 74.3% of the time for unknown Sharp-tailed Grouse (Table A1, Supplemental Figure A4a). Average membership probabilities for Lesser Prairie-Chicken individuals were relatively high (0.91), while average membership probabilities for plains Sharp-tailed Grouse and unknown Sharp-tailed Grouse individuals were intermediate to low (0.68, and 0.67, respectively; Table A2). Average membership probabilities for plains Sharp-tailed Grouse were 0.10 Lesser Prairie-Chicken, 0.68 plains Sharp-tailed Grouse, and 0.22 unknown Sharp-tailed Grouse (Table A2). Average membership probabilities for unknown Sharp-tailed Grouse were 0.14 Lesser Prairie-Chicken, 0.19 plains Sharp-tailed Grouse, and 0.67 unknown Sharp-tailed Grouse (Table A2). In general, from this model on 3 populations using 15 covariates, Lesser Prairie-Chickens occupied their own morphological spaces while plains Sharp-tailed Grouse and unknown Sharp-tailed Grouse mostly occupied their own space, however there was some overlap (Figure A4b). Our Random Forests model evaluating 15 covariates on three populations correctly classified 92.8% of Lesser Prairie-Chickens, 79.4% of plains Sharp-tailed Grouse, and 83.4% of unknown Sharp-tailed Grouse (Table A3). The three morphological factors with the highest importance in the expanded morphological covariates Random Forests model were the wing cord length to tail length ratio (1.00), tail length (0.81), and the tail length to total head length ratio (0.80; Table A7).

**Microsatellite Genotyping**—We did not collect microsatellite loci from Lesser Prairie-Chicken samples; therefore, we do not have any microsatellite loci results including Lesser Prairie-Chickens.

**Whole Genome Resequencing**—Using a DAPC analysis on low-resolution whole genome resequencing single nucleotide variants (SNVs) data, the model correctly predicted 100.0% of Lesser Prairie-Chicken, 66.7% of Columbian Sharp-tailed Grouse, 33.3% of plains Sharp-tailed Grouse, and 76.9% of unknown Sharp-tailed Grouse (Table 1, Figure A1b). The average membership probabilities for Lesser Prairie-Chicken individuals was high (1.00), while average membership probabilities for Columbian Sharp-tailed Grouse, plains Sharp-tailed Grouse, and unknown Sharp-tailed Grouse individuals were low (0.56, 0.39, and 0.49, respectively; Table A2). In general, Lesser Prairie-Chicken occupied a unique principal components space while Columbian, plains, and unknown Sharp-tailed Grouse generally shared a principal components space (Figure A2b). Our Random Forests model evaluating 453 single nucleotide variants across all sequenced individuals correctly classified 0.0% of Lesser Prairie-Chickens, 50.0% of Columbian Sharp-tailed Grouse, 33.3% of plains Sharp-tailed Grouse, and 53.9% of unknown Sharp-tailed Grouse (Table A3). Population genetic statistics for single nucleotide variants are reported in Table 4.

**
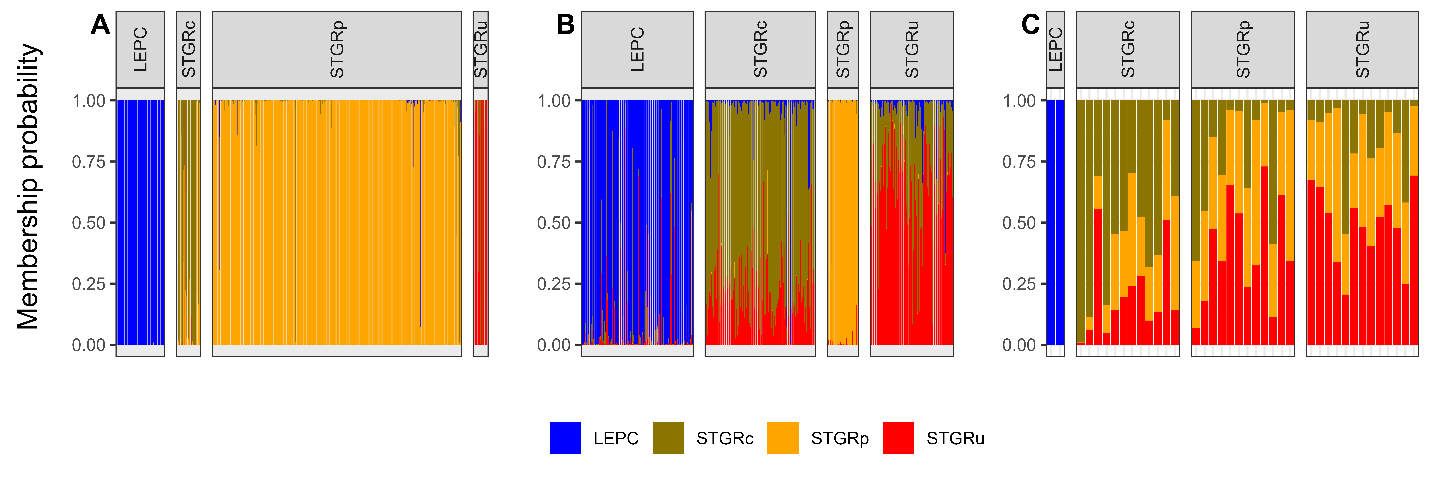
Figure A1.** Membership probability (admixture) plots for discriminant analysis of principal components for habitat characteristics (**A**), morphological characteristics (mass, wing cord length, tarsus + longest toe length, tail length, and all pairwise combinations; **B**), single nucleotide variants (SNVs; **C**), and for Lesser Prairie-Chicken (LEPC), Columbian Sharp-tailed Grouse (STGRc), plains Sharp-tailed Grouse (STGRp), and unknown Sharp-tailed Grouse subspecies (STGRu). Membership probability plot depicts the proportion of assignment to each population, with different colors representing the proportion of each population in each individual. Facets represent the original population of each individual (morphology and SNVs) or observations (habitat). Habitat data (eBird observation locations) from Colorado, Idaho, Kansas, Montana, Nebraska, Nevada, New Mexico, North Dakota, Oklahoma, South Dakota, Texas, Utah, Washington, and Wyoming, 2010–2023. Morphological data were collected in Kansas and Colorado (LEPC; 2013–2017), Idaho and Washington (STGRc; 2005–2013), and Wyoming (STGRp, 2019; STGRu, 2017–2019). Single nucleotide variant data were collected on individuals sampled from Kansas (LEPC, 2013), Idaho (STGRc, 2018), and Wyoming (STGRp 2019; STGRu, 2017–2018).


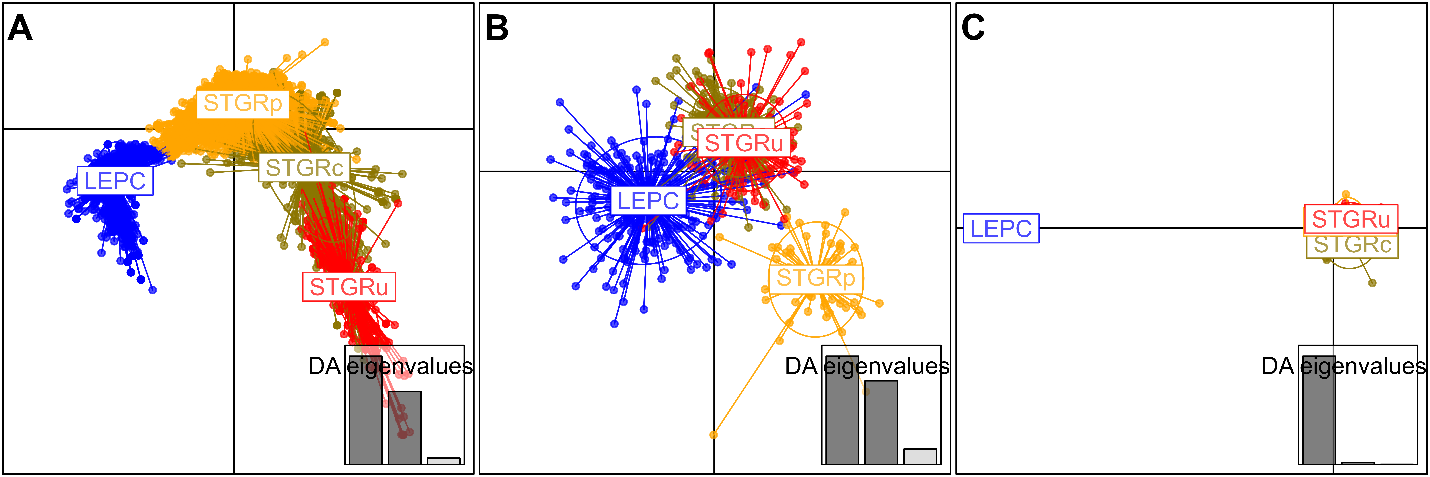


**Figure A2.** Principal components plots from discriminant analysis of principal components for habitat characteristics (**A**), morphological characteristics (mass, wing cord length, tarsus + longest toe length, tail length, and all pairwise combinations; **B**), and 453 single nucleotide variants (SNVs; **C**) for Lesser Prairie-Chicken (LEPC; blue), Columbian Sharp-tailed Grouse (STGRc; greenish-gold), plains Sharp-tailed Grouse (STGRp; yellow-orange), and unknown Sharp-tailed Grouse subspecies (STGRu; red). Habitat data (eBird observation locations) from Colorado, Idaho, Kansas, Montana, Nebraska, Nevada, New Mexico, North Dakota, Oklahoma, South Dakota, Texas, Utah, Washington, and Wyoming, 2010–2023. Morphological data were collected in Kansas and Colorado (LEPC; 2013–2017), Idaho and Washington (STGRc; 2005–2013), and Wyoming (STGRp, 2019; STGRu, 2017–2019). Single nucleotide variant data were collected on individuals sampled from Kansas (LEPC, 2013), Idaho (STGRc, 2018), and Wyoming (STGRp 2019; STGRu, 2017–2018). Points represent individuals (morphology and SNVs) or individual observations (habitat).


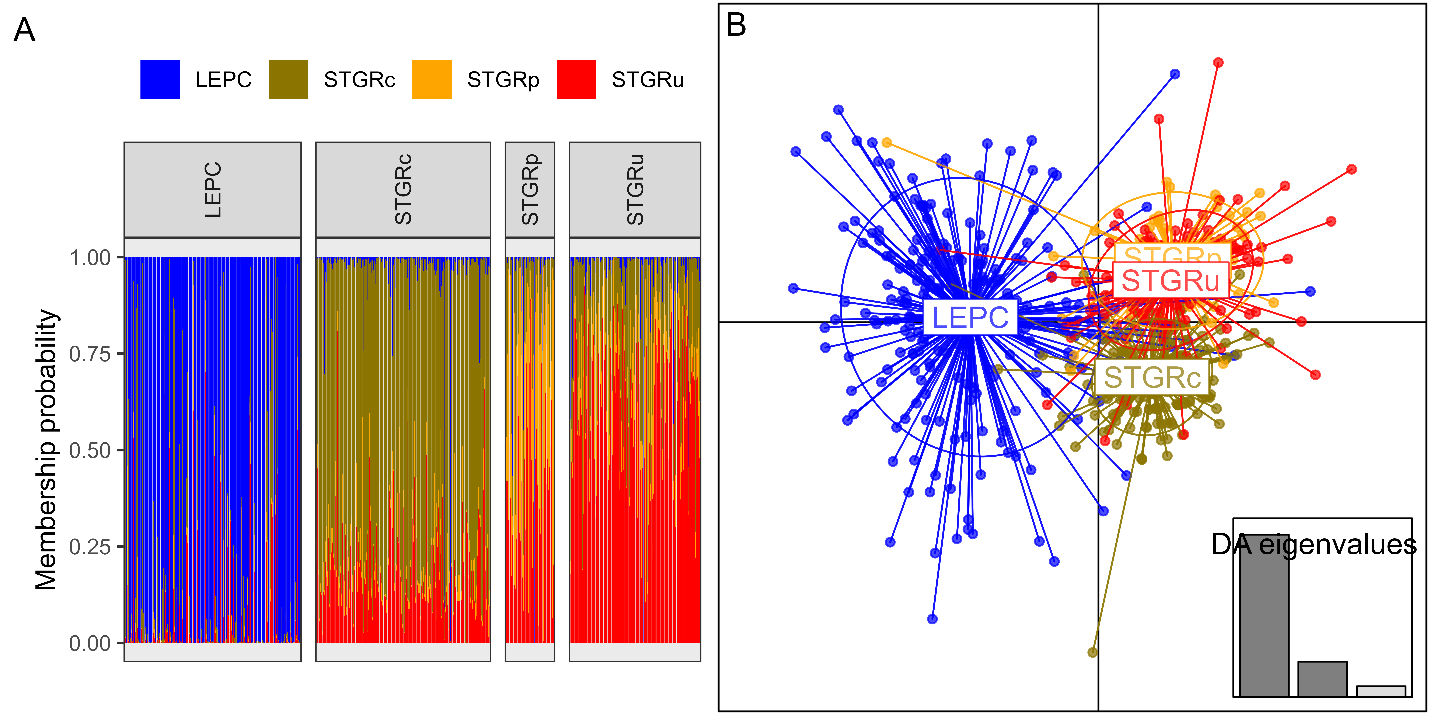


**Figure A3.** Membership probability (admixture) plot (**A**) and principal components plot (**B**) from discriminant analysis of principal components (DAPC) for morphological characteristics excluding mass (wing cord length, tarsus + longest toe length, tail length, and all pairwise combinations) for Lesser Prairie-Chicken (LEPC), Columbian Sharp-tailed Grouse (STGRc), plains Sharp-tailed Grouse (STGRp), and unknown Sharp-tailed Grouse (STGRu). Morphological data were collected in Kansas and Colorado (LEPC; 2013–2017), Idaho and Washington (STGRc; 2005–2013), and Wyoming (STGRp, 2019; STGRu, 2017–2019).


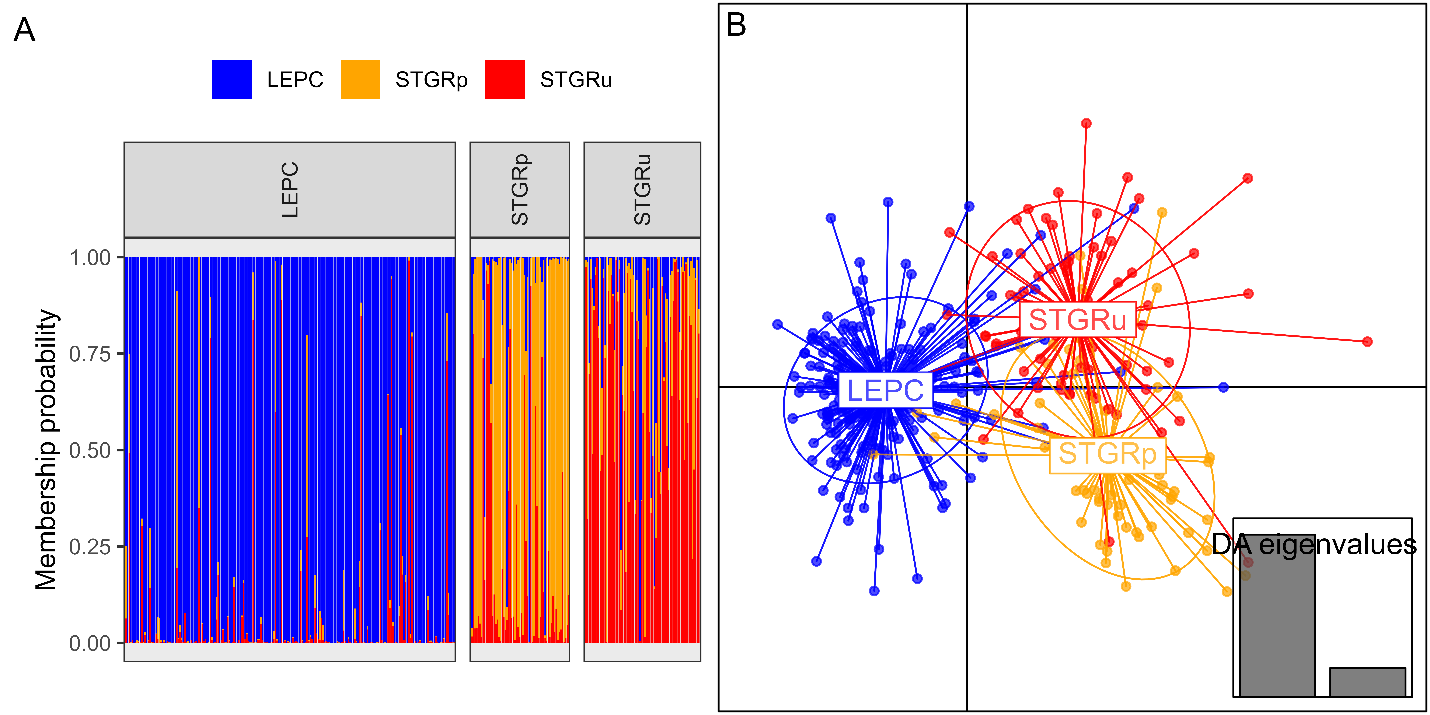


**Figure A4.** Membership probability (admixture) plot (**A**) and principal components plot (**B**) from discriminant analysis of principal components (DAPC) for expanded morphological characteristics (total head length, culmen length, wing cord length, tarsus + longest toe length, tail length, and all pairwise combinations) for Lesser Prairie-Chicken (LEPC), plains Sharp-tailed Grouse (STGRp), and unknown Sharp-tailed Grouse (STGRu). Morphological data were collected in Kansas and Colorado (LEPC; 2013–2017) and Wyoming (STGRp, 2019; STGRu, 2017–2019).

**Table A1.** Number of variables, sample sizes, and assignment probabilities of discriminant analysis of principal components on habitat characteristics, morphological characteristic, and single nucleotide variants (SNVs) for populations of Lesser Prairie-Chicken (LEPC), Columbian Sharp-tailed Grouse (STGRc), plains Sharp-tailed Grouse (STGRp), and a population with unknown subspecific status of Sharp-tailed Grouse in south-central Wyoming (STGRu). Habitat data (eBird observation locations) from Colorado, Idaho, Kansas, Montana, Nebraska, Nevada, New Mexico, North Dakota, Oklahoma, South Dakota, Texas, Utah, Washington, and Wyoming, 2010–2023. Morphological data were collected in Kansas and Colorado (LEPC; 2013–2017), Idaho and Washington (STGRc; 2005–2013), and Wyoming (STGRp, 2019; STGRu, 2017–2019). Single nucleotide variant data were collected on individuals sampled from Kansas (LEPC, 2013), Idaho (STGRc, 2018), and Wyoming (STGRp 2019; STGRu, 2017–2018). Empty cells (—) represent populations that were not included in that particular analysis.

|  |  | Sample size | | | | Assignment probability | | | |
| --- | --- | --- | --- | --- | --- | --- | --- | --- | --- |
| Analyses | Number of variables | LEPC | cSTGR | pSTGR | uSTGR | LEPC | cSTGR | pSTGR | uSTGR |
| Habitat | 22 | 1,576 | 812 | 7,951 | 509 | 1.00 | 0.66 | 0.98 | 0.84 |
| Morphology | 6^a^ | 222 | 219 | 63 | 165 | 0.84 | 0.95 | 0.41 | 0.77 |
|  | 10^b^ | 222 | 219 | 63 | 165 | 0.84 | 0.93 | 0.97 | 0.82 |
|  | 15^c^ | 210 | — | 63 | 74 | 0.93 | — | 0.71 | 0.74 |
| SNVs | 453 | 2 | 12 | 12 | 13 | 1.00 | 0.67 | 0.33 | 0.77 |

^a^Morphological analysis using tail length (mm), wing cord length (mm), tarsus + longest toe length (mm), and all pairwise comparisons.

^b^Morphological analysis using mass (g), tail length (mm), wing cord length (mm), tarsus + longest toe length (mm), and all pairwise comparisons

^c^Morphological analysis using tail length (mm), wing cord length (mm), tarsus + longest toe length (mm), culmen length, total head length, and all pairwise combinations

**Table A2.** Mean and median average membership probability of each individual assigned to each population of prairie-grouse evaluated using a discriminant analysis of principal components based on habitat characteristics, morphological characteristics, and single nucleotide variants (SNVs) for 4 populations of prairie-grouse: Lesser Prairie-Chicken (LEPC), Columbian Sharp-tailed Grouse (STGRc), plains Sharp-tailed Grouse (STGRp), and a population of Sharp-tailed Grouse with unknown subspecific status (STGRu) in south-central Wyoming. Habitat data (eBird observation locations) from Colorado, Idaho, Kansas, Montana, Nebraska, Nevada, New Mexico, North Dakota, Oklahoma, South Dakota, Texas, Utah, Washington, and Wyoming, 2010–2023. Morphological data were collected in Kansas and Colorado (LEPC; 2013–2017), Idaho and Washington (STGRc; 2005–2013), and Wyoming (STGRp, 2019; STGRu, 2017–2019). Single nucleotide variant data were collected on individuals sampled from Kansas (LEPC, 2013), Idaho (STGRc, 2018), and Wyoming (STGRp 2019; STGRu, 2017–2018).

|  |  | Mean (median) average membership probability of individuals | | | |
| --- | --- | --- | --- | --- | --- |
| Analyses | Population | LEPC | STGRc | STGRp | STGRu |
| Habitat | LEPC | 1.00 (1.00) | 0.00 (0.00) | 0.00 (0.00) | 0.00 (0.00) |
|  | STGRc | <0.01 (0.00) | 0.66 (0.96) | 0.25 (0.01) | 0.09 (0.00) |
|  | STGRp | 0.01 (0.00) | 0.02 (0.00) | 0.98 (1.00) | <0.01 (0.00) |
|  | STGRu | <0.01 (0.00) | 0.15 (0.00) | <0.01 (0.00) | 0.85 (1.00) |
| Morphological^a^ | LEPC | 0.80 (0.99) | 0.11 (0.01) | 0.02 (0.00) | 0.07 (0.01) |
|  | STGRc | 0.03 (0.01) | 0.74 (0.78) | 0.05 (0.03) | 0.18 (0.16) |
|  | STGRp | 0.05 (0.01) | 0.19 (0.14) | 0.40 (0.38) | 0.36 (0.35) |
|  | STGRu | 0.04 (0.01) | 0.25 (0.21) | 0.12 (0.08) | 0.59 (0.64) |
| Morphological^b^ | LEPC | 0.81 (0.99) | 0.10 (0.01) | 0.01 (0.00) | 0.08 (0.00) |
|  | STGRc | 0.04 (0.01) | 0.75 (0.80) | 0.00 (0.00) | 0.21 (0.18) |
|  | STGRp | 0.01 (0.00) | 0.01 (0.00) | 0.96 (1.00) | 0.02 (0.00) |
|  | STGRu | 0.04 (0.01) | 0.30 (0.27) | 0.00 (0.00) | 0.65 (0.70) |
| Morphological^c^ | LEPC | 0.91 (1.00) | — | 0.02 (0.00) | 0.06 (0.00) |
|  | STGRp | 0.10 (0.01) | — | 0.68 (0.91) | 0.22 (0.07) |
|  | STGRc | 0.14 (0.04) | — | 0.19 (0.07) | 0.67 (0.76) |
| SNVs | LEPC | 1.00 (1.00) | 0.00 (0.00) | 0.00 (0.00) | 0.00 (0.00) |
|  | STGRc | 0.00 (0.00) | 0.56 (0.54) | 0.24 (0.24) | 0.20 (0.14) |
|  | STGRp | 0.00 (0.00) | 0.23 (0.12) | 0.39 (0.36) | 0.38 (0.34) |
|  | STGRu | 0.00 (0.00) | 0.16 (0.09) | 0.35 (0.33) | 0.49 (0.52) |

^a^Morphological analysis using tail length (mm), wing cord length (mm), tarsus + longest toe length (mm), and all pairwise comparisons.

^b^Morphological analysis using mass (g), tail length (mm), wing cord length (mm), tarsus + longest toe length (mm), and all pairwise comparisons

^c^Morphological analysis using tail length (mm), wing cord length (mm), tarsus + longest toe length (mm), culmen length, total head length, and all pairwise combinations

**Table A3.** Pairwise comparisons and classification error (%) of Random Forests classification for four populations of prairie-grouse based on habitat characteristics, morphological characteristics, and single nucleotide variants. Populations evaluated were Lesser Prairie-Chicken (LEPC), Columbian Sharp-tailed Grouse (STGRc), plains Sharp-tailed Grouse (STGRp), and unknown Sharp-tailed Grouse (STGRu). Habitat data (eBird observation locations) from Colorado, Idaho, Kansas, Montana, Nebraska, Nevada, New Mexico, North Dakota, Oklahoma, South Dakota, Texas, Utah, Washington, and Wyoming, 2010–2023. Morphological data were collected in Kansas and Colorado (LEPC; 2013–2017), Idaho and Washington (STGRc; 2005–2013), and Wyoming (STGRp, 2019; STGRu, 2017–2019). Single nucleotide variant data were collected on individuals sampled from Kansas (LEPC, 2013), Idaho (STGRc, 2018), and Wyoming (STGRp 2019; STGRu, 2017–2018).

| Analysis |  | LEPC | STGRc | STGRp | STGRu | Classification error (%) |
| --- | --- | --- | --- | --- | --- | --- |
| Habitat | LEPC | 1,576 | 0 | 0 | 0 | 0.0 |
|  | STGRc | 0 | 757 | 54 | 1 | 6.8 |
|  | STGRp | 1 | 7 | 7943 | 0 | 0.1 |
|  | STGRu | 0 | 8 | 1 | 500 | 1.8 |
| Morphological^a^ | LEPC | 194 | 15 | 0 | 13 | 12.6 |
|  | STGRc | 14 | 193 | 0 | 12 | 11.9 |
|  | STGRp | 2 | 0 | 60 | 1 | 4.8 |
|  | STGRu | 11 | 13 | 1 | 140 | 15.2 |
| Morphological^b^ | LEPC | 194 | 14 | 0 | 14 | 12.6 |
|  | STGRc | 11 | 193 | 3 | 12 | 11.9 |
|  | STGRp | 4 | 5 | 33 | 21 | 47.6 |
|  | STGRu | 10 | 14 | 15 | 126 | 23.6 |
| Morphological^c^ | LEPC | 194 | – | 3 | 12 | 7.2 |
|  | STGRp | 9 | – | 50 | 4 | 20.6 |
|  | STGRu | 5 | – | 7 | 62 | 16.2 |
| Single nucleotide variants | LEPC | 0 | 1 | 0 | 1 | 100.0 |
|  | STGRc | 0 | 6 | 3 | 3 | 50.0 |
|  | STGRp | 0 | 3 | 5 | 4 | 58.3 |
|  | STGRu | 0 | 4 | 3 | 6 | 53.8 |

^a^Morphological analysis using tail length (mm), wing cord length (mm), tarsus + longest toe length (mm), and all pairwise comparisons.

^b^Morphological analysis using mass (g), tail length (mm), wing cord length (mm), tarsus + longest toe length (mm), and all pairwise comparisons

^c^Morphological analysis using tail length (mm), wing cord length (mm), tarsus + longest toe length (mm), culmen length, total head length, and all pairwise combinations

**Table A4.** Standardized variable importance for Random Forests model predicting prairie-grouse populations based on habitat characteristics at observed locations (eBird), 2010–2023. Prairie-grouse populations evaluated were Lesser Prairie-Chicken, Columbian Sharp-tailed Grouse, plains Sharp-tailed Grouse, and a population of Sharp-tailed Grouse with unknown subspecific status. Variable importance values were standardized so the top variable equals 1 and the remaining variables are proportions derived by dividing by the top variable (Doherty et al. 2018). PRISM represents 30-year average annual climate data (PRISM Climate Group 2014); RAP represents rangeland analysis platform data (Robinson et al. 2019, Alred et al. 2021, Jones et al. 2021); and NLCD represents the National Land Cover Database from 2011, 2013, 2016, 2019, and 2021 (Jin et al. 2019).

| Variable | Importance Value |
| --- | --- |
| Mean maximum annual temperature (PRISM) | 1.00 |
| Percent cover of shrubs (RAP) | 0.55 |
| Mean annual precipitation (PRISM) | 0.21 |
| Terrain ruggedness index | 0.16 |
| Emergent wetland (NLCD) | 0.14 |
| Perennial herbaceous vegetation biomass (RAP) | 0.11 |
| Heat load index | 0.11 |
| Percent cover of litter (RAP) | 0.10 |
| Annual herbaceous vegetation biomass (RAP) | 0.09 |
| Percent cover of perennial herbaceous vegetation (RAP) | 0.08 |
| Water (NLCD) | 0.07 |
| Percent cover of annual herbaceous vegetation (RAP) | 0.07 |
| Canopy cover of coniferous forests (RAP) | 0.07 |
| Topographic position index | 0.07 |
| Canopy cover of deciduous forests (RAP) | 0.07 |
| Croplands (NLCD) | 0.06 |
| Unclassified forests canopy cover (RAP) | 0.05 |
| Canopy cover of all forest types (RAP) | 0.05 |
| Percent bare ground (RAP) | 0.05 |
| Pasture lands (NLCD) | 0.05 |
| Anthropogenic development (NLCD) | 0.04 |
| Canopy cover of mixed forests (RAP) | 0.02 |

**Table A5.** Standardized variable importance for Random Forests model predicting prairie-grouse populations based on morphological characteristics (mass (g), tail length (mm), wing cord length (mm), tarsus + longest toe length (mm) [tarsus + toe length], and all pairwise comparisons). Prairie-grouse populations evaluated were Lesser Prairie-Chicken (2013–2017; Kansas and Colorado), Columbian Sharp-tailed Grouse (2005–2013; Idaho and Washington), plains Sharp-tailed Grouse (2019; Wyoming), and a population of Sharp-tailed Grouse with unknown subspecific status (2017–2019; Wyoming). Variable importance values were standardized so the top variable equals 1 and the remaining variables are proportions derived by dividing by the top variable (Doherty et al. 2018).

| Variable | Importance value |
| --- | --- |
| Wing cord length to tail length ratio | 1.00 |
| Tarsus + toe length | 0.91 |
| Wing cord length to tarsus + toe length ratio | 0.76 |
| Tail length | 0.71 |
| Tail length to mass ratio | 0.66 |
| Tail length to tarsus + toe length ratio | 0.53 |
| Mass | 0.52 |
| Tarsus + toe length to mass ratio | 0.40 |
| Wing cord length:mass | 0.40 |
| Wing cord length | 0.16 |

**Table A6.** Standardized variable importance for Random Forests model predicting prairie-grouse populations based on morphological characteristics (tail length (mm), wing cord length (mm), tarsus + longest toe length (mm) [tarsus + toe length], and all pairwise comparisons). Prairie-grouse populations evaluated were Lesser Prairie-Chicken (2013–2017; Kansas and Colorado), Columbian Sharp-tailed Grouse (2005–2013; Idaho and Washington), plains Sharp-tailed Grouse (2019; Wyoming), and a population of Sharp-tailed Grouse with unknown subspecific status (2017–2019; Wyoming). Variable importance values were standardized so the top variable equals 1 and the remaining variables are proportions derived by dividing by the top variable (Doherty et al. 2018).

| Variable | Importance value |
| --- | --- |
| Wing cord length to tail length ratio | 1.00 |
| Tarsus + toe length | 0.93 |
| Tail length | 0.74 |
| Wing cord length to tarsus + toe length ratio | 0.72 |
| Tail length to tarsus +toe length ratio | 0.71 |
| Wing cord length | 0.41 |

**Table A7.** Standardized variable importance for Random Forests model predicting prairie-grouse populations based on morphological characteristics (tail length (mm), wing cord length (mm), tarsus + longest toe length (mm; tarsus + toe), culmen length, total head length, and all pairwise combinations). Prairie-grouse populations evaluated were Lesser Prairie-Chicken (2013–2017; Kansas and Colorado), plains Sharp-tailed Grouse (2019; Wyoming), and a population of Sharp-tailed Grouse with unknown subspecific status (2017–2019; Wyoming). Variable importance values were standardized so the top variable equals 1 and the remaining variables are proportions derived by dividing by the top variable (Doherty et al. 2018).

| **Variable** | **Importance value** |
| --- | --- |
| Wing cord length to tail length ratio | 1.00 |
| Tail length | 0.81 |
| Tail length to total head length ratio | 0.80 |
| Tail length to culmen length ratio | 0.63 |
| Tail length to tarsus +toe length ratio | 0.46 |
| Total head length | 0.45 |
| Total head length to tarsus +toe length ratio | 0.43 |
| Wing cord length | 0.34 |
| Wing cord length to total head length ratio | 0.32 |
| Tarsus +toe length | 0.32 |
| Total head length to culmen length ratio | 0.30 |
| Wing cord length to tarsus +toe length ratio | 0.26 |
| Culmen length | 0.20 |
| Culmen length to tarsus +toe length ratio | 0.19 |
| Wing cord length to culmen length ratio | 0.17 |
