# Supplementary material for: Using Habitat, Morphological, and Genetic Characteristics to Delineate the Subspecies of Sharp‐Tailed Grouse in South‐Central Wyoming
Source: Ecol Evol. 2025 May 12;15(5):e71429. doi: 10.1002/ece3.71429 (PMC12068902; doi:10.1002/ece3.71429)
Supplement: Supplementary file 2 — Table S1. Table S2. Table S3. Figure S1. [file ECE3-15-e71429-s002.docx]

**Supplemental Materials Table S1.** Standardized variable importance for Random Forests model predicting prairie-grouse populations based on habitat characteristics at observed locations (eBird), 2010–2023. Prairie-grouse populations evaluated were Columbian Sharp-tailed Grouse, plains Sharp-tailed Grouse, and a population of Sharp-tailed Grouse with unknown subspecific status. Variable importance values were standardized so the top variable equals 1 and the remaining variables are proportions derived by dividing by the top variable (Doherty et al. 2018). PRISM represents 30-year average annual climate data (PRISM Climate Group 2014); RAP represents annual Rangeland Analysis Platform data (Robinson et al. 2019, Alred et al. 2021, Jones et al. 2021); NLCD represents the National Land Cover Database from 2011, 2013, 2016, 2019, and 2021 (Jin et al. 2019); canopy cover of different forest types was derived from both NLCD and RAP data layers.

| **Variable** | **Importance value** |
| --- | --- |
| Percent cover of shrubs (RAP) | 1.00 |
| Terrain ruggedness index | 0.27 |
| Heat load index | 0.22 |
| Topographic position index | 0.17 |
| Percent cover of perennial herbaceous vegetation (RAP) | 0.16 |
| Canopy cover of deciduous forest (NLCD and RAP) | 0.15 |
| Mean annual precipitation (PRISM) | 0.13 |
| Percent cover of annual herbaceous vegetation (RAP) | 0.12 |
| Annual herbaceous vegetation biomass (RAP) | 0.11 |
| Croplands (NLCD) | 0.11 |
| Mean annual maximum temperature (PRISM) | 0.10 |
| Biomass of perennial herbaceous vegetation (RAP) | 0.10 |
| Canopy cover of unclassified forest (NLCD and RAP) | 0.09 |
| Canopy cover of all forest types (NLCD and RAP) | 0.09 |
| Canopy cover of coniferous forest (NLCD and RAP) | 0.09 |
| Percent cover of litter (RAP) | 0.09 |
| Anthropogenic development (NLCD) | 0.06 |
| Water (NLCD) | 0.06 |
| Pasture lands (NLCD) | 0.05 |
| Emergent wetlands (NLCD) | 0.05 |
| Percent bare ground (RAP) | 0.04 |
| Canopy cover of mixed forests (RAP) | 0.03 |

**Supplemental Materials Table S2.** Standardized variable importance for Random Forests model predicting prairie-grouse populations based on morphological characteristics (mass (g), tail length (mm), wing cord length (mm), tarsus + longest toe length (mm) [tarsus + toe length], and all pairwise comparisons). Prairie-grouse populations evaluated were Columbian Sharp-tailed Grouse (2005–2013; Idaho and Washington), plains Sharp-tailed Grouse (2019; Wyoming), and a population of Sharp-tailed Grouse with unknown subspecific status (2017–2019; Wyoming). Variable importance values were standardized so the top variable equals 1 and the remaining variables are proportions derived by dividing by the top variable (Doherty et al. 2018).

| **Variable** | **Importance value** |
| --- | --- |
| Tarsus + toe length | 1.00 |
| Wing cord length to tarsus + toe length ratio | 0.84 |
| Mass | 0.50 |
| Tarsus + toe length to mass ratio | 0.42 |
| Wing cord length to mass ratio | 0.39 |
| Tail length to mass ratio | 0.21 |
| Tail length to tarsus + toe length ratio | 0.14 |
| Wing cord length to tail length ratio | 0.11 |
| Wing cord length | 0.11 |
| Tail length | 0.07 |

**Supplemental Materials Table S3.** Standardized variable importance for Random Forests model predicting prairie-grouse populations based on morphological characteristics (tail length (mm), wing cord length (mm), tarsus + longest toe length (mm) [tarsus + toe length], and all pairwise comparisons). Prairie-grouse populations evaluated were Columbian Sharp-tailed Grouse (2005–2013; Idaho and Washington), plains Sharp-tailed Grouse (2019; Wyoming), and a population of Sharp-tailed Grouse with unknown subspecific status (2017–2019; Wyoming). Variable importance values were standardized so the top variable equals 1 and the remaining variables are proportions derived by dividing by the top variable (Doherty et al. 2018).

| **Variable** | **Importance value** |
| --- | --- |
| Tarsus + toe length | 1.00 |
| Wing cord length to tarsus + toe length ratio | 0.78 |
| Wing cord length | 0.40 |
| Tail length to tarsus + toe length ratio | 0.33 |
| Wing cord length to tail length ratio | 0.24 |
| Tail length | 0.20 |

**
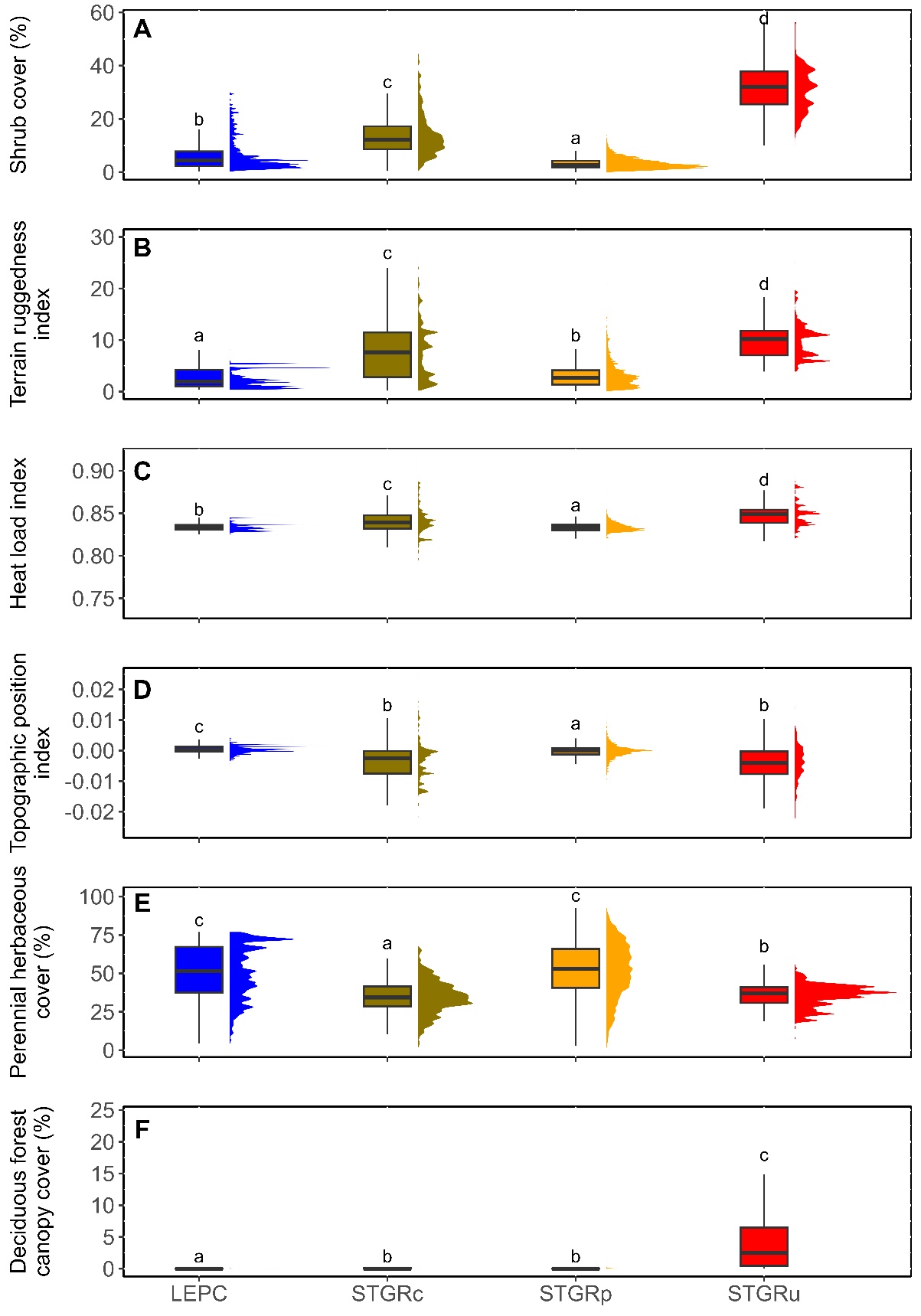
**

**Supplemental Materials Figure S1.** Comparison of raw habitat characteristics at eBird checklist locations across four populations of grouse: Lesser Prairie-Chicken (LEPC), Columbian Sharp-tailed Grouse (STGRc), plains Sharp-tailed Grouse (STGRp), and a Sharp-tailed Grouse with unknown subspecific status (STGRu) in south-central Wyoming (2010–2023). Habitat characteristics include the top six most important variables from a Random Forests classification model (see Supplemental Materials Table S1). Habitat characteristics include percent cover of shrubs (**A**), terrain ruggedness index (**B**), heat load index (**C**), topographic position index (**D**), percent cover of perennial herbaceous vegetation (**E**), and percent canopy cover of deciduous forests (**F**). Superscript letters above each boxplot represent statistical differences calculated using a Kruskal-Wallis rank sum test, where populations with the same letter did not differ from each other.
